# A Unified Operator Resolution of Quadratic Bond Orders

**DOI:** 10.1021/acs.jpca.6c04675

**Published:** 2026-10-10

**Authors:** Dariusz W. Szczepanik

**Affiliations:** K. Gumiński Department of Theoretical Chemistry, Faculty of Chemistry, 37799 Jagiellonian University, Gronostajowa 2, Kraków 30-387, Poland

## Abstract

Quadratic bond-order indices provide compact measures of chemical covalency, but their scalar form conceals the orbital channels contributing to a bond. We present a common operator framework for closed-shell, open-shell, and correlated one-particle densities in a fixed orthonormal atomic partition. Bond indices are defined as traces of positive-semidefinite atom-pair operators, recovering the Wiberg and per-spin Mayer forms in the respective single-determinant limits. Spectral resolution, or diagonal projection onto a complete common spinless basis, yields non-negative orbital weights summing to the trace of the corresponding operator. For correlated densities, occupation-scaled cumulative spectral layers define a distinct one-particle extension, generally different from direct quadratic evaluation. The additional criterion is population consistency: in the exact spectral construction, the layer weights reconstruct the supplied density, while the associated quadratic population partition retains its orbital and atomic population marginals. These populations are stationary under the resulting Markov transition matrix without requiring an idempotent physical density. The exact construction requires no near-degeneracy threshold and is continuous with respect to the supplied density within the fixed atomic representation. Implemented in runEDDB and illustrated on a chemically diverse set of main-group, transition-metal, and actinide systems, the framework provides a bond-by-bond and symmetry-resolved description of covalency and localization.

## Graphical abstract

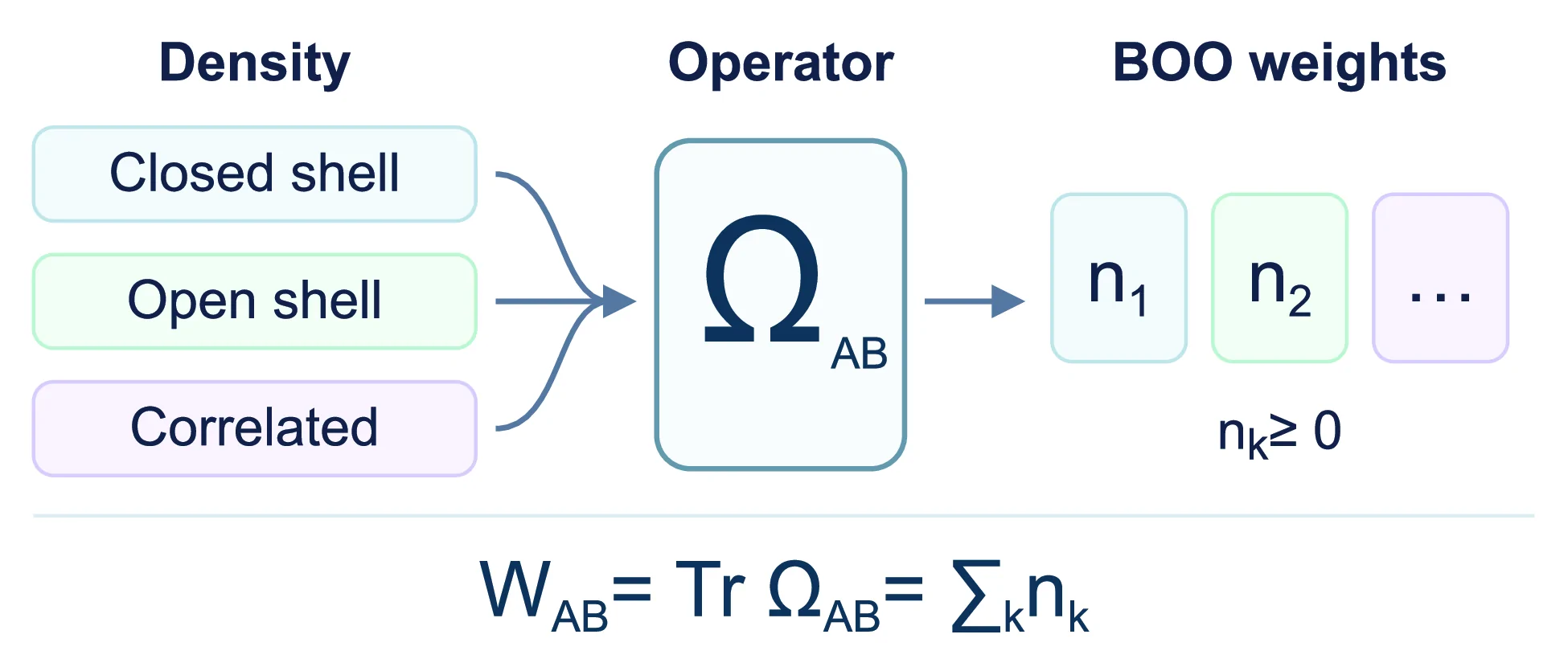

## 1 Introduction

The chemical bond is the organizing abstraction of chemistry, and bond order is the number by which computational chemistry most often puts that abstraction on a quantitative scale. Its role is no longer confined to retrospective bonding interpretation. Bond-order and closely related pair-population measures enter molecular graph construction, automated detection of reactive events in *ab initio* molecular dynamics,^1^ quantum-mechanically augmented force-field parametrization,^2^ transition-metal spin-state and bond-valence metrics,^3^ and aromaticity or electron-delocalization analyses in molecules and extended systems.^4–6^ Even local correlation methods, where the object being compressed is a pair-correlation function rather than a bond-order index, rely on the same chemical economy of atom-pair and orbital-pair domains.^7^ The practical appeal is always the same: a complicated electronic structure is reduced to chemically legible pair channels.

This success also exposes a weakness. The conventional bond order is usually reported as a scalar attached to an atom pair, but the chemistry of that pair is rarely scalar. A C–C bond may contain separable σ and π contributions; a transition-metal or actinide bond may carry δ components, and in suitable high-angular-momentum cases even φ components; an aromatic ring may be delocalized in one symmetry channel and localized or antiaromatic in another. At the same time, the algebra used to evaluate the index changes with the electronic structure model: the closed-shell Wiberg form, the open-shell per-spin Mayer form, and correlated-density generalizations are usually treated as separate prescriptions. Thus, two limitations appear together: the scalar index hides the orbital channels that give a bond its character, and the evaluation of the same chemical quantity is not written uniformly across restricted, unrestricted, open-shell, and correlated descriptions.

The purpose of this work is to keep the familiar quadratic Wiberg–Mayer covalency index, but to give it an operator resolution. Once an atomic partition and an atom-respecting orthonormal one-particle representation have been fixed, the two-center density block of an atom pair can be used to construct a positive semidefinite bond-order density operator. Its trace is the bond index, and its spectral resolution—or, in the open-shell case, a trace-preserving projection onto a common spinless atom-pair basis—defines bond-order orbitals (BOOs) with non-negative weights that add exactly to the index. In this qualified sense the index acquires the spectral structure normally associated with an observable, without pretending that the result is independent of the underlying atomic partition.

The scalar index from which we start is the quadratic Wiberg–Mayer bond order, although its historical development is broader than the two names suggest. Early electron-pair formulations by Okada and Fueno and the TEV index of Giambiagi and coworkers introduced closely related pair-population measures and addressed their extension to nonorthogonal bases.^8–11^ Wiberg established the familiar orthonormal-basis index in semiempirical theory,^12^ whereas Mayer developed the *ab initio* SCF formulation and subsequently clarified its metric, valence, and open-shell properties in a sequence of papers and an addendum.^13–18^ Both forms are implemented in widely used analysis programs, including Multiwfn^19^ and natural bond orbital software,^20^ and have fuzzy-atom and real-space counterparts.^21^ The quantitative theory of bond order is in large measure due to Mayer: beyond the index itself, his effective atomic orbitals,^22^ chemical Hamiltonian,^23^ and bond-order-weighted energy components^24^ established bond order as a theoretically grounded descriptor rather than a mere Lewis count. Writing **γ** for the spin-summed 1-RDM, **S** for the overlap matrix, and *A*, *B* for the basis functions on two atoms, the Mayer index is the bilinear functional

1 $$B_{AB}=\underset{\mu \in A}{\sum }\underset{\nu \in B}{\sum }{\left(\gamma S\right)}_{\mu \nu }{\left(\gamma S\right)}_{\nu \mu }$$

which in an orthonormal basis (**S** = **I**) reduces to the squared Frobenius norm of the off-diagonal density block, $B_{AB}=\underset{\mu \in A,\nu \in B}{\sum }\gamma_{\mu \nu }^{2}$ . We refer to this common orthonormal-basis quantity as the quadratic Wiberg–Mayer bond-covalency index.

For a closed-shell single Slater determinant and a fixed orthonormal atomic partition, the quadratic index in eq 1 also has a population-fluctuation interpretation. Its exchange contraction coincides with the delocalization index,^25–27^

2 $$\begin{array}{ll} B_{AB} & =\delta \left(A,B\right)=-2\mathrm{cov}\left(N_{A},N_{B}\right),\\ \mathrm{cov}\left(N_{A},N_{B}\right) & =\left\langle N_{A}N_{B}\right\rangle -\left\langle N_{A}\right\rangle \left\langle N_{B}\right\rangle \end{array}$$

where *N*_*A*_ denotes the electron-number operator of atomic domain *A*. For an open-shell determinant, the corresponding exchange contraction uses the separate spin blocks, as in eq 8. The real-space counterpart is the delocalization index of Fradera, Austen, and Bader.^28^

For a correlated wave function, the covariance in eq 2 depends on the full exchange–correlation contribution to the pair density: the exchange term constructed from the correlated spin-resolved 1-RDM and the connected two-particle cumulant. The cumulant vanishes for a single Slater determinant, but it is not the entire exchange–correlation contribution.^29,30^ Consequently, direct quadratic evaluation from the correlated spin blocks retains the exchange contribution but does not, in general, reproduce the full population-fluctuation index. The equality between the quadratic bond order and the covariance-based index must therefore be restricted to the single-determinant case. The consequences of using first- rather than second-order density matrices, and of choosing different atomic partitions, were examined by Matito et al.^31^ in their comparison of QTAIM delocalization indices and fuzzy-atom bond orders at correlated levels. Their analysis illustrates that these choices can retain different features of the bonding description.

A negative covariance-based delocalization index, when it occurs, indicates positive covariance between the atomic populations; it is not a mathematical failure of that definition. The non-negative one-particle construction developed here has a different target. It extends the projector-based quadratic population allocation while preserving its population marginals and a stationary conditional-probability description, rather than reconstructing the full two-particle covariance. This connection to orbital communication theory motivates the spectral-layer construction discussed below.^32–34^

Operator-based treatments of bond populations have precedents. Carbó-Dorca and Bultinck formulated Hermitian operators within the LCAO MO framework to obtain atomic and bond populations from first- and second-order densities, respectively.^35^ The construction developed here focuses on a density-dependent positive semidefinite bond-order operator defined on a fixed atom-pair one-particle space. Its trace recovers the quadratic Wiberg–Mayer index in the appropriate determinant limits and defines the chosen spectral-layer extension for correlated densities. The operator admits an orthonormal spectral resolution; alternatively, its diagonal projection onto a complete common spinless atom-pair basis preserves the trace without, in general, reconstructing the full operator.

This construction is formally analogous to Löwdin’s natural-orbital decomposition of the molecular 1-RDM,^36^ but the analogy must be qualified. Bond-order orbitals are properties of the density relative to the chosen atomic partition, not partition-independent quantum observables. In a nondegenerate spectral sector an individual BOO is unique up to its overall sign, whereas within a degenerate sector only the spectral projector, the associated subspace, and its total weight are uniquely defined. The invariant content of the resolution therefore lies in its spectrum, spectral subspaces, and trace rather than in a privileged orientation of individual orbitals inside a degenerate space.

It is this operator content, rather than a particular orbital representation, that generalizes. Once the quadratic bond order is expressed as the trace of a positive bond-order density, the closed-shell, open-shell, and correlated cases become different constructions of the operator to be traced, while Hermiticity, non-negativity, and the trace sum rule are retained throughout.

The need for a unified operator formulation becomes clearest beyond the closed-shell idempotent limit. For a closed-shell determinant the spin-summed density is duodempotent and the Wiberg form applies directly. For a spin-polarized determinant, the same quadratic covalency must instead be evaluated as the per-spin Mayer sum to avoid artificial *αβ* cross terms. For a correlated state the problem is more fundamental: the 1-RDM has fractional occupations, while the two-particle cumulant causes exchange-based and fluctuation-based definitions of bond order, equivalent at the single-determinant level, to diverge.^29,30^

Previous correlated extensions have consequently addressed different physical targets. Mayer introduced a pseudospin-density construction designed to recover the appropriate dissociation behavior of correlated singlet wave functions,^37^ whose properties were later examined in detail by Cooper, Ponec, and Karadakov.^38^ Full delocalization indices, by contrast, retain the cumulant contribution of the 2-RDM and therefore quantify a different object from a quadratic functional of the 1-RDM. These alternatives are not competing notations for one universally defined correlated bond order; they preserve different aspects of the single-determinant concept.

The present work deliberately adopts a narrower, operator-compatible target: a positive, trace-conserving one-particle continuation of the quadratic Wiberg–Mayer covalency index, with exact closed-shell and open-shell determinant limits and a common orbital resolution. Related density-based descriptors—including multicenter delocalization indices,^4^ effective oxidation states,^39^ and the natural adaptive orbitals of Pendás and Francisco^40^—address complementary aspects of chemical bonding. Our aim here is to provide a single operator resolution that can be evaluated consistently from closed-shell, open-shell, and correlated one-particle densities while preserving positivity and an exact trace sum rule.

Energy-based analyses provide a complementary perspective on chemical bonding. Early work by Ruedenberg examined the physical origin of bond energies,^41^ while interaction-energy decomposition schemes were developed by Kitaura and Morokuma^42^ and by Ziegler and Rauk.^43^ Commonly used EDA formulations distinguish electrostatic, Pauli-repulsion, and orbital-interaction contributions.^44^ Combining the extended transition state scheme with natural orbitals for chemical valence (ETS–NOCV) further resolves the deformation density and associated orbital-interaction energies into bonding channels.^45,46^ These fragment-based energy and deformation-density decompositions complement the present analysis, which resolves a quadratic atom-pair covalency index from the supplied molecular density rather than partitioning an interaction energy.

A complementary tradition seeks not a number but a set of localized bond orbitals: natural bond orbitals (NBO),^20^ intrinsic bond orbitals (IBO),^47^ adaptive natural density partitioning (AdNDP),^48^ and the classical Foster–Boys and Pipek–Mezey localizations.^49,50^ These methods differ in their criteria and algorithms, but each selects a particular orbital representation of the occupied space, whose projector is itself invariant under rotations among the occupied orbitals. For saturated molecules the result is usually a near-canonical Lewis structure, whereas for conjugated, aromatic, or electron-deficient systems several localized representations of comparable quality can coexist, which is the orbital-space counterpart of competing resonance structures and motivates resonance-weighted descriptions.^51^ The present construction takes a different route. Once the atomic partition and the atom-respecting orthonormal representation have been fixed, it characterizes the bonding channels directly from the atom-pair block of **γ**, without selecting a gauge in the occupied space. The singular values and singular subspaces are invariants of this block; individual singular vectors are unique up to sign in nondegenerate sectors and up to unitary rotations within degenerate sectors. The resulting resolution is therefore independent of any orbital-localization criterion and remains well-defined precisely where localized-orbital pictures become nonunique.

The operator construction requires a fixed decomposition of the chosen one-particle space into mutually orthogonal atomic subspaces, but does not require a particular atomic-orbital construction. Orthogonality allows the atom-pair blocks to be treated by ordinary Hermitian eigenproblems and singular value decompositions, without an explicit AO overlap metric. The atomic partition nevertheless remains part of the definition: invariance under rotations within fixed atomic subspaces does not imply identical numerical indices for different choices of those subspaces.

We use natural atomic orbitals (NAOs),^20,52^ whose density-derived core–valence–Rydberg organization is convenient for both population analysis and the shell bookkeeping of the optional angular-momentum classification. In the separated-atom limit with spherically symmetric atomic densities, this construction recovers the corresponding atomic natural-orbital description, up to rotations within degenerate subspaces. The untruncated NAO set spans the same finite one-particle space as the original linearly independent AO basis, retaining the weakly occupied components of a correlated density rather than projecting them onto a minimal valence space. This choice also keeps the bond-order analysis in the atomic representation used by the runEDDB workflow. Minimal/valence reductions (NMB/NVB) are considered only when explicitly stated.^53^

Intrinsic atomic orbitals (IAOs)^47^ and quasi-atomic orbitals (QAOs)^54,55^ provide alternative chemically motivated atomic representations. The original IAO construction supplies a minimal atom-associated basis that exactly represents the occupied SCF space. For a general correlated 1-RDM, any population outside such a minimal space must also be represented by retaining an appropriate complement. QAO formulations explicitly distinguish bonding and correlating spaces and have been developed for strongly correlated MCSCF wave functions; their use is therefore not excluded by the present approach.

The choice of NAOs is thus practical and interpretive, not a claim of mathematical necessity or superiority over IAO/QAO analyses. The bond-order operator and spectral-layer construction can be applied in other mutually orthogonal atomic partitions, provided that the retained space and any truncation are specified. Their algebraic properties then hold within the chosen representation, while the numerical bond indices may reflect the different atomic partition.

The density notation is fixed as follows. The spin-summed one-particle density matrix is denoted by **D**. When spin resolution is required, **P** ^α^ and **P** ^β^ denote the corresponding one-spin density matrices, so that **D** = **P** ^α^ + **P** ^β^. For a single Slater determinant the spin blocks are idempotent projectors, (**P** ^ς^)^2^ = **P** ^ς^, whereas a closed-shell spin-summed density is duodempotent, **D** ^2^ = 2**D**. Thus, eq 3 below is the closed-shell Wiberg form of the same quadratic index, while eq 8 is its open-shell per-spin Mayer form. The dummy spin label is denoted by ς ∈ {α, β}, to distinguish it from the singular values σ_*i*_ and the σ symmetry channel.

Section 2 develops the closed-shell construction; Section 3 the open-shell projection scheme; Section 4 the correlated extension; Section 5 the optional symmetry labeling; Section 6 the computational protocol; and Section 7 the numerical illustrations.

## 2 The Bond-Order Block and Its Orbitals (Closed Shell)

With the density and basis conventions fixed above, let *A* and *B* denote two disjoint atomic subspaces of the chosen orthonormal representation, and let **D**_*AB*_ be the corresponding rectangular (*n*_*A*_ × *n*_*B*_) off-diagonal block of the spin-summed 1-RDM. The closed-shell Wiberg form of the quadratic Wiberg–Mayer bond-covalency index is

3 $$W_{AB}=\underset{i\in A}{\sum }\underset{j\in B}{\sum }D_{ij}^{2}={∥D_{AB}∥}_{F}^{2}$$

which, for a closed-shell single-determinant density (**D** ^2^ = 2**D**), recovers the standard shared-pair count (*W* = 1 for H_2_, *W*  ≈ 1.44 for an aromatic C–C bond). Because **D**_*AB*_ is rectangular it is not diagonalizable, but it embeds naturally in the symmetric coupling matrix on *A* ∪ *B*,

4 $$M=\left(\begin{array}{ll} 0 & D_{AB}\\ D_{AB}^{T} & 0 \end{array}\right)\in R^{\left(n_{A}+n_{B})\times (n_{A}+n_{B}\right)}$$

The spectrum of **M** is symmetric about zero: for every singular value σ_*i*_ of **D**_*AB*_ (with left/right singular vectors **u**_*i*_ ∈ *A*, **v**_*i*_ ∈ *B*) there is an eigenpair

5 $$Mw_{i}^{\pm }=\pm \sigma_{i}w_{i}^{\pm },,w_{i}^{\pm }=\frac{1}{\sqrt{2}}\left(u_{i},\pm v_{i}\right)$$

This paired spectrum is closely related to the orthogonal-projector partitioning of the molecular 1-RDM developed by Vanfleteren, Van Neck, Bultinck, Ayers, and Waroquier.^56,57^ Their analysis explicitly identifies paired bonding and antibonding modes of the interatomic contribution and provides a stockholder construction of the atomic projectors. For fixed mutually orthogonal atomic subspaces, the Hermitian sum of the two interatomic density blocks has the same block structure as eq 4. This established coupling structure is the starting point here. Our focus is the positive quadratic bond-order operator defined below and its per-spin and occupation-scaled spectral-layer extensions, rather than a new construction of the atomic projectors. The population-normalization criterion underlying the layer extension is developed in Section 4.

The positive branch $w_{i}^{+}$ is the bonding atom-pair combination, whereas the negative branch is its antibonding mirror. We refer to the eigenvectors of the positive branch as *bond-order orbitals* (BOOs).^58^ For a nondegenerate singular value, $w_{i}^{+}$ is defined up to its overall sign. If several singular values are degenerate, only the corresponding positive-eigenvalue subspace and its projector are unique; individual BOOs may be rotated unitarily within that subspace. Such a rotation does not affect the operator, its spectrum, or the total weight of the degenerate sector.

The closed-shell bond-order density operator is
6

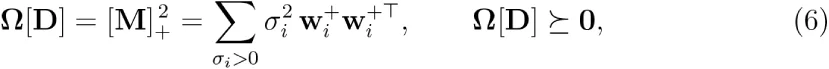

where [**M**]_+_ denotes the positive spectral part of **M**. Its trace is

7 $$\mathrm{Tr}\Omega \left[D\right]=\underset{\sigma_{i}>0}{\sum }\sigma_{i}^{2}={∥D_{AB}∥}_{F}^{2}=W_{AB}$$

Thus, the squared positive eigenvalues provide an exact, non-negative, and additive resolution of the quadratic bond order.^58,59^ In the simplest examples, H_2_ has one nonzero BOO with weight 1, whereas an aromatic C–C bond is resolved predominantly into a σ component with weight close to 1 and a π component with weight close to 0.44. The construction requires only a symmetric eigensolver, and the ± pairing of **M** supplies the left and right singular subspaces at negligible cost for the small (*n*_*A*_ + *n*_*B*_) atom-pair blocks involved.

## 3 Open-Shell Single-Determinant Densities: The Spinless Projection Scheme

The closed-shell expression ([Disp-formula eq3]) relies on the spin-summed density being duodempotent. For a spin-polarized single-determinant density,

$$D=P^{\alpha }+P^{\beta }$$

the spin blocks are individually idempotent, but the spin-summed density is not duodempotent in general. Direct insertion of **D** into the closed-shell Frobenius norm would produce

$${∥D_{AB}∥}_{F}^{2}={∥P_{AB}^{\alpha }∥}_{F}^{2}+{∥P_{AB}^{\beta }∥}_{F}^{2}+2\underset{i\in A}{\sum }\underset{j\in B}{\sum }P_{ij}^{\alpha }P_{ij}^{\beta }$$

thereby adding spin cross terms that are not part of the open-shell Mayer index. The correct open-shell determinant form of the same quadratic Wiberg–Mayer bond-covalency index is the per-spin Mayer sum

8 $$W_{AB}=2\underset{\varsigma \in \left\{\alpha ,\beta \right\}}{\sum }\underset{i\in A}{\sum }\underset{j\in B}{\sum }{\left(P_{ij}^{\varsigma }\right)}^{2},,{\left(P^{\varsigma }\right)}^{2}=P^{\varsigma }$$

The factor of 2 converts the one-spin density convention to the usual spin-summed bond-order convention and makes ([Disp-formula eq8]) reduce exactly to ([Disp-formula eq3]) at the closed-shell limit.

Each spin channel has its own atom-pair coupling matrix **M** ^ς^, built from $P_{AB}^{\varsigma }$ as in ([Disp-formula eq4]), and hence its own positive bonding branch. We define the one-spin bond-order density

9 $$\Omega^{\varsigma }=\underset{\sigma_{i}^{\varsigma }>0}{\sum }{\left(\sigma_{i}^{\varsigma }\right)}^{2}w_{i}^{\varsigma ,+}w_{i}^{\varsigma ,+T},,\mathrm{Tr}\Omega^{\varsigma }={∥P_{AB}^{\varsigma }∥}_{F}^{2}$$

which is symmetric positive semidefinite. The spin-summed open-shell bond-order density is then

10 $$\Omega =2\left(\Omega^{\alpha }+\Omega^{\beta }\right),,\mathrm{Tr}\Omega =W_{AB}$$

This operator contains the correct per-spin Mayer trace and no *αβ* cross terms.

### 3.1 Choice of Orbital Representation

The operator in eq 10 admits several valid orbital representations. Retaining the separate α- and β-BOOs preserves explicit spin information, and their contributions, multiplied by the factor of 2 in that equation, sum exactly to *W*_*AB*_. Their spatial parts need not be mutually orthogonal between spin channels, but this does not invalidate the spin-resolved description.

Alternatively, direct diagonalization of the combined operator gives an orthonormal spectral resolution,

11 $$\begin{array}{ll} & \Omega =\underset{j}{\sum }\omega_{j}z_{j}z_{j}^{T},\\ & \omega_{j}\geq 0,,\underset{j}{\sum }\omega_{j}=W_{AB} \end{array}$$

This is an exact decomposition of the full bond-order operator and also recovers the closed-shell resolution. Neither separate spin orbitals nor direct diagonalization needs to be replaced on mathematical grounds.

The common spinless representation used here has a different practical purpose: it separates the definition of the spatial coupling modes from the evaluation of their spin-dependent weights. The modes are obtained from the linear total-density coupling

$$M=M^{\alpha }+M^{\beta },,D_{AB}=P_{AB}^{\alpha }+P_{AB}^{\beta }$$

whereas their weights are evaluated using the quadratic, spin-resolved operator **Ω**. For a fixed total density and atomic partition, the same spatial frame can therefore be retained while the spin-resolved contributions to its occupations are examined. This provides a common atom-pair display without changing the underlying bond-order definition.

The nonzero modes of **M** also retain the paired singular-vector structure of eq 4: each has equal total squared amplitude on the two atomic subspaces, with the positive and negative branches differing in their relative sign. This is a property of the coupling representation, not an assertion that the physical bond is unpolarized. Eigenvectors of the combined operator **Ω** need not retain this paired form.

Let $Q=\left(m_{1},...,m_{n_{A}+n_{B}}\right)$ be a complete orthonormal eigenbasis of **M**,

$$MQ=Q\Lambda ,,Q^{T}Q=I$$

The positive branch gives the usual spinless bonding combinations. The negative and null branches must also be retained because, away from the closed-shell limit, **Ω** need not have its support entirely in the positive branch of **M**.

The effective occupations in this common frame are

12 $$\begin{array}{ll} n_{k} & ={\left(Q^{T}\Omega Q\right)}_{kk}=\left\langle m_{k}\right|\Omega \left|m_{k}\right\rangle \geq 0,\\ \overset{n_{A}+n_{B}}{\underset{k=1}{\sum }}n_{k} & =\mathrm{Tr}\Omega =W_{AB} \end{array}$$

Positivity follows from **Ω** 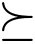 **0**, and the sum rule follows from the completeness of **Q**. At the closed-shell limit, $P^{\alpha }=P^{\beta }=\frac{1}{2}D$ , the negative and null branches carry zero occupation, and the positive-branch weights reduce to $n_{k}=\sigma_{k}^{2}$ .

For a general open-shell density, however, **Q** ^T^ **ΩQ** need not be diagonal. The projected occupations therefore preserve the trace, but do not reconstruct the full operator unless its off-diagonal elements in this frame vanish. Direct diagonalization of **Ω** retains that information in its spectral modes; the common spinless display instead assigns weights to modes defined by the total-density coupling. Within a degenerate eigenspace of **M**, individual projected occupations can depend on the chosen basis, whereas their sum over the complete eigenspace is invariant.

These representations serve complementary purposes: separate spin BOOs retain explicit spin resolution, eigenvectors of **Ω** provide its exact spectral decomposition, and the common spinless frame reports spin-resolved bond-order weights in the spatial coupling modes of the total density. A three-function analytic example in the Supporting Information works out all three representations for one open-shell determinant and shows explicitly that the projected weights preserve the trace while an off-diagonal element of **Ω** in the coupling frame remains. The last choice is a reporting convention, not an additional physical approximation to *W*_*AB*_ or a claim of universal diagnostic superiority.

## 4 Correlated Wave Functions: Occupation-Scaled Cumulative Spectral Layers

Nonidempotency does not prevent direct evaluation of the quadratic bond-order functional. For a correlated spin-summed density, the quantity ${∥D_{AB}∥}_{F}^{2}$ remains non-negative and admits the operator resolution of Section 2; the corresponding direct per-spin construction is likewise well-defined for fractional occupations. Both retain their respective single-determinant limits. Positivity, an exact orbital sum rule, and recovery of these limits therefore do not, by themselves, distinguish the spectral-layer extension developed here from direct evaluation.

The additional criterion adopted here is consistency with the normalization of the underlying quadratic population partition. For an exact spectral decomposition, occupation-weighted cumulative layers reconstruct the supplied 1-RDM, while their quadratic contributions retain the population-normalization identity associated with idempotent densities. This criterion connects the present construction to the stationary information-channel formulation of Szczepanik and Mrozek.^33^ It motivates a particular one-particle extension, rather than establishing a unique definition of correlated bond order. Because the bond-order functional is nonlinear, its layer-weighted evaluation generally differs from its direct evaluation on the reconstructed density. Accordingly, trace preservation refers throughout to the orbital resolution of the constructed bond-order operator, not to equality between the direct and layer-defined correlated indices.

Other correlated definitions of bond order preserve different aspects of the single-determinant concept. Exchange-based and population-fluctuation-based definitions, equivalent at the single-determinant level, need not coincide when the two-particle cumulant is nonzero.^29,30^ Mayer’s pseudospin-density construction addresses the dissociation of correlated singlet wave functions into open-shell fragments,^37^ and its properties were subsequently examined by Cooper, Ponec, and Karadakov.^38^ The present layer construction is neither a replacement for these alternatives nor an evaluation of the full exchange-correlation contribution to the pair-density delocalization index. Its purpose is to extend the projector-based population analysis while retaining a non-negative bond-order operator and a consistent orbital resolution.

We use the cumulative spectral-layer decomposition introduced in refs 33 and 59. In the exact construction, the natural orbitals are ordered by decreasing occupation, and each group *G*_*l*_ spans the complete eigenspace of one distinct occupation ν_*l*_. A nondegenerate occupation therefore defines a one-orbital group. In the notation below, *n̅*_*l*_ = ν_*l*_ for these exact eigenspaces. Grouping near-degenerate occupations and replacing them by a common mean is a numerical approximation, not a requirement of the mathematical definition. Let

13 $$\Pi_{l}=\underset{k\in G_{l}}{\sum }\left|\phi_{k}\right\rangle \left\langle \phi_{k}\right|,,\Pi_{\leq l}=\underset{m\leq l}{\sum }\Pi_{m},,\Pi_{\leq l}^{2}=\Pi_{\leq l}$$

be the spectral projector onto the *l*th occupation shell and the corresponding cumulative spectral projector. The objects inserted into the bond-order functional are the occupation-scaled cumulative density layers

14 $$\begin{array}{ll} P_{l}^{\left(\eta \right)} & =\eta \Pi_{\leq l}=\eta \underset{k\in \underset{m\leq l}{\cup }G_{m}}{\sum }\left|\phi_{k}\right\rangle \left\langle \phi_{k}\right|\\ \eta & =2\;\mathrm{for a spin‐summed density},,\eta =1\;\mathrm{for one spin block} \end{array}$$

Only the cumulative projectors **Π**_≤*l*_ are idempotent in the usual sense. The scaled layers $P_{l}^{\left(\eta \right)}$ obey instead ${\left(P_{l}^{\left(\eta \right)}\right)}^{2}=\eta P_{l}^{\left(\eta \right)}$ ; they are idempotent only in the one-spin convention, and are duodempotent in the closed-shell spin-summed convention. In what follows the superscript *(η)* is omitted when the convention is clear.

For each layer we build **Ω**[**P**_*l*_] from the off-diagonal block of **P**_*l*_ in the same way as in ([Disp-formula eq9]). The correlated bond-order density is then assembled from the occupation-weighted increments

15 $$\Omega =\underset{l}{\sum }w_{l}\left(\Omega \left[P_{l}\right]-\Omega \left[P_{l-1}\right]\right),,w_{l}=\left\{ \begin{array}{ll} \frac{1}{2}{\mathrm{n̅}}_{l}, & \mathrm{for a spin‐summed density}\left(\eta =2\right),\\ {\mathrm{n̅}}_{l}, & \mathrm{for one spin block}\left(\eta =1\right), \end{array}\right.$$

with **P**_0_ = **0**. The trace Tr **Ω** defines the layer-consistent quadratic bond-covalency index, and the spinless BOOs and their effective occupations are obtained from the projection formula ([Disp-formula eq12]). The point of the construction is not to interpolate between recipes, but to retain the same algebraic safeguards—the exact single-determinant limit, non-negativity, and representation invariance—when the physical 1-RDM is no longer idempotent.

**Proposition 1** (Exact idempotent limit). *If the density supplied to the layer construction is a single-determinant density—idempotent per spin, (* **P** ^*ς*^ *)^2^ =* **P** ^*ς*^, *or duodempotent in the closed-shell spin-summed convention,* **D** *^2^ = 2* **D** *—then* ([Disp-formula eq15]) *reduces identically to the single-determinant bond-order operators of* Sections 2 *–* [Sec sec3]. *In particular, Tr* **Ω** *= W*_*AB*_ *: the Wiberg index (* [Disp-formula eq3] *) for a closed shell and the per-spin Mayer index* ([Disp-formula eq8]) *for an open shell. The reduction is an algebraic identity, not an approximation.*

*Proof*. Consider first a single density channel: the spin-summed density **D** for a closed shell, or one spin block **P** ^ς^ otherwise. In the single-determinant limit the natural occupations are either η or 0, with η = 2 for the closed-shell spin-summed density and η = 1 for a one-spin density. Thus, every occupied natural orbital carries the same occupation η, and every virtual natural orbital carries occupation zero. Processing the orbitals one layer at a time is sufficient for the proof; the QD grouping only collects orbitals of exactly equal occupation and therefore cannot change the telescoping result.

For every occupied layer the weight in ([Disp-formula eq15]) is *w*_*l*_ = 1, because $w_{l}=\frac{1}{2}{\mathrm{n̅}}_{l}=1$ in the closed-shell convention and *w*_*l*_ = *n̅*_*l*_ = 1 in the one-spin convention. For every virtual layer the weight is zero. Hence the layer sum telescopes over the occupied space,

$$\Omega =\overset{N_{\mathrm{occ}}}{\underset{k=1}{\sum }}\left(\Omega \left[P_{k}\right]-\Omega \left[P_{k-1}\right]\right)=\Omega \left[P_{N_{\mathrm{occ}}}\right]-\Omega \left[0\right]=\Omega \left[P_{N_{\mathrm{occ}}}\right]$$

because **Ω**[**0**] = **0**. The terminal layer is the full single-determinant density of the channel,

$$P_{N_{\mathrm{occ}}}=\eta \underset{\mathrm{occ}}{\sum }\left|\phi_{m}\right\rangle \left\langle \phi_{m}\right|$$

namely **D** for a closed shell and **P** ^ς^ for one spin block.

Therefore, **Ω** = **Ω**[**D**] in the closed-shell case, and

$$\mathrm{Tr}\Omega ={∥D_{AB}∥}_{F}^{2}=W_{AB}$$

by ([Disp-formula eq7]). For an unrestricted single determinant, the same argument applied separately to the α and β spin blocks gives **Ω**[**P** ^α^] and **Ω**[**P** ^β^], which recombine through ([Disp-formula eq10]) as

$$\Omega =2\left(\Omega \left[P^{\alpha }\right]+\Omega \left[P^{\beta }\right]\right)$$

Its trace is therefore the per-spin Mayer index ([Disp-formula eq8]). An earlier form of this telescoping argument was first sketched in refs 33,59 .

**Proposition 2** (Non-negativity). *For any admissible spin-summed or one-spin 1-RDM supplied to the layer construction,* **Ω** *is positive semidefinite. Consequently every projected occupation in* ([Disp-formula eq12]) *satisfies n_k_ ≥ 0.*

*Proof*. Let

$$A_{l}=\Omega \left[P_{l}\right],,A_{0}=0$$

where the superscript *(η)* on the occupation-scaled layer $P_{l}^{\left(\eta \right)}$ is suppressed. Equation 15 can then be written as

$$\Omega =\overset{K}{\underset{l=1}{\sum }}w_{l}\left(A_{l}-A_{l-1}\right)$$

Abel summation gives the equivalent form

16 $$\Omega =\overset{K}{\underset{l=1}{\sum }}\left(w_{l}-w_{l+1}\right)\Omega \left[P_{l}\right],,w_{K+1}\equiv 0$$

The natural occupations are sorted in nonincreasing order, and therefore the layer weights *w*_*l*_ are also nonincreasing: *w*_*l*_ – *w*_*l*+1_ ≥ 0. For every *l*, the operator **Ω**[**P**_*l*_] is positive semidefinite by construction, because it is built as in ([Disp-formula eq9]) from squared singular values and outer products of normalized atom-pair orbitals. Equation 16 is therefore a non-negative linear combination of positive semidefinite operators, so **Ω** is positive semidefinite. Finally,

$$n_{k}=\left\langle m_{k}\right|\Omega \left|m_{k}\right\rangle \geq 0$$

for every vector of the complete spinless atom-pair basis used in ([Disp-formula eq12]).

### 4.1 Population Normalization and Markov Stationarity

The spectral layers also preserve a population-normalization property that motivated the original information-channel construction.^33^ Let **X** denote the spin-summed density **D** for η = 2, or either spin block **P** ^α^ or **P** ^β^ for η = 1, with *N*_*X*_ = Tr **X** > 0. Consider its exact distinct positive natural occupations ν_1_ > ··· > ν_*L*_ > 0 and set ν_*L*+1_ = 0. The occupation-gap weights and the corresponding density reconstruction are

17 $$\begin{array}{ll} a_{l} & =\frac{\nu_{l}-\nu_{l+1}}{\eta }\geq 0,\\ X & =\overset{L}{\underset{l=1}{\sum }}a_{l}P_{l}^{\left(\eta \right)},,P_{l}^{\left(\eta \right)}=\eta \Pi_{\leq l} \end{array}$$

These are the same coefficients *a*_*l*_ = *w*_*l*_ – *w*_*l*+1_ that enter eq 16; they follow from the spectrum of the supplied density, rather than from an additional normalization fitted to the bond index.

Define the layer-weighted quadratic population kernel

18 $$K_{\mu \nu }=\overset{L}{\underset{l=1}{\sum }}a_{l}{|{\left(P_{l}^{\left(\eta \right)}\right)}_{\mu \nu }|}^{2}$$

This kernel is symmetric and entrywise non-negative. Since ${\left(P_{l}^{\left(\eta \right)}\right)}^{2}=\eta P_{l}^{\left(\eta \right)}$ , its row sums satisfy

19 $$\begin{array}{ll} \underset{\nu }{\sum }K_{\mu \nu } & =\underset{l}{\sum }a_{l}{\left[{\left(P_{l}^{\left(\eta \right)}\right)}^{2}\right]}_{\mu \mu }\\ & =\eta \underset{l}{\sum }a_{l}{\left(P_{l}^{\left(\eta \right)}\right)}_{\mu \mu }=\eta X_{\mu \mu } \end{array}$$

The sum includes the diagonal and all intra- and interatomic contributions. It is not restricted to bonded pairs or to components retained for printing.

On the support where *X*_*μμ*_ > 0, introduce

20 $$T_{\nu |\mu }=\frac{K_{\mu \nu }}{\eta X_{\mu \mu }},,p_{\mu }=\frac{X_{\mu \mu }}{N_{X}}$$

Equation 19 gives ∑_ν_ *T*_ν|μ_ = 1. Moreover, symmetry of the kernel implies

21 $$\begin{array}{ll} p_{\mu }T_{\nu |\mu } & =\frac{K_{\mu \nu }}{\eta N_{X}}=p_{\nu }T_{\mu |\nu },\\ \underset{\mu }{\sum }p_{\mu }T_{\nu |\mu } & =p_{\nu } \end{array}$$

Thus, the orbital population distribution of the supplied density is stationary under the associated Markov transition matrix, although **X** itself need not be idempotent.

The same population accounting carries over to atomic domains. Writing *K*_*AB*_ = ∑_μ∈*A*_ ∑_ν∈*B*_ *K*_*μν*_ and *N*_*A*_ = ∑_μ∈*A*_ *X*_*μμ*_ gives

$$\underset{B}{\sum }K_{AB}=\eta N_{A}$$

with the one-center term *B* = *A* included. For distinct atoms, *K*_*AB*_ is the trace of the layer-defined bond-order operator for the selected density channel; the open-shell index combines the two spin-channel traces with the factor of 2 in eq 10.

Direct quadratic evaluation instead gives ∑_ν_|*X*_*μν*_|^2^ = (**X** ^2^)_*μμ*_, which generally differs from η*X*_*μμ*_. The additional property retained by the layer construction is therefore the normalization of the projector-based population allocation, not merely non-negativity of individual bond indices. Stationarity alone does not uniquely determine a correlated bond-order definition, nor does this auxiliary Markov description represent physical electron dynamics.

The identities above refer to exact occupation eigenspaces. If nearby occupations are replaced by their group means, the same identities hold for the corresponding group-averaged density; this numerical approximation must be distinguished from the exact spectral construction.

### 4.2 Exact Degeneracies and Numerical Grouping

The exact spectral construction requires no near-degeneracy threshold. Its behavior when a degeneracy is lifted can be seen by rewriting the occupation-gap sum as an integral. For an admissible density channel 0 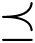 **X** 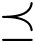 η**I**, define the spectral projector

$$\Pi_{>t}\left[X\right]=\underset{\nu_{l}>t}{\sum }\Pi_{l},,0\leq t\leq \eta$$

Here *t* is an integration variable, not an adjustable grouping parameter. Denoting the assembled layer operator by **Ω**_layer_[**X**], eq 16 becomes

22 $$\Omega_{\mathrm{layer}}\left[X\right]=\frac{1}{\eta }\int_{0}^{\eta }\Omega \left[\eta \Pi_{>t}\left[X\right]\right]dt$$

Indeed, on each interval (ν_*l*+1_, ν_*l*_) the projector is **Π**_≤*l*_, and integration gives the coefficient (ν_*l*_ – ν_*l*+1_)/η of the corresponding cumulative layer.

Equation 22 also establishes continuity of the exact operator in a fixed finite-dimensional atomic representation. If a sequence of admissible densities converges to **X**, its spectral projectors converge for every *t* outside the finite set of eigenvalues of **X**. The associated bond-order operators then converge as well, because taking the positive spectral part and squaring are continuous matrix operations. The integrands are uniformly bounded, since the projectors have norm at most one and η is fixed. Integration therefore gives convergence of **Ω**_layer_ and of its trace.

Consequently, lifting an occupation degeneracy does not introduce a finite jump in the exact bond index. The additional cumulative projectors created by the splitting enter over occupation intervals whose widths vanish as the degeneracy is restored. The individual natural orbitals within the splitting subspace may change substantially, but this does not produce a discontinuity of the assembled operator. Continuity does not imply differentiability at every degeneracy or a unique continuous choice of individual orbitals.

Numerical grouping must be distinguished from this exact result. Replacing nearby occupations by their group means evaluates the construction for a group-averaged density rather than for the original density. A finite grouping tolerance can therefore introduce threshold-dependent changes when group membership changes. These are numerical approximation effects, not an intrinsic ambiguity of the exact spectral definition; their magnitude must be assessed separately. The invariance statements below refer to exact occupation eigenspaces.

**Proposition 3** (Universality within a fixed atomic representation). *After the orthonormal atomic representation and the atomic partition have been fixed, the layer construction is a well-defined functional of the one-particle density. It is invariant under any unitary rotation within an occupation-degenerate natural-orbital subspace and under any reordering of orbitals inside such a subspace. Moreover, for any spin-unpolarized density with* $P^{\alpha }=P^{\beta }=\frac{1}{2}D$ , *the spin-summed* (*η = 2*) *construction and the per-spin* (*η = 1*) *construction followed by* ([Disp-formula eq10]) *give the same bond-order density operator* **Ω**. *Consequently W*_*AB*_ *= Tr* **Ω** *and its BOO resolution are identical for restricted and unrestricted treatments whenever they supply the same spin-unpolarized 1-RDM.*

*Proof*. The first statement follows directly from the spectral-projector form of the construction. If a set of natural orbitals spans an occupation-degenerate subspace, any unitary rotation inside that subspace changes the individual orbitals but leaves the projector

$${\mathrm{\Pi ̂}}_{l}=\underset{k\in G_{l}}{\sum }\left|\phi_{k}\right\rangle \left\langle \phi_{k}\right|$$

unchanged. Hence the cumulative projector Π̂_≤*l*_ = ∑*m*_≤_ *_l_* Π̂_*m*_, the occupation-scaled layer ${\mathrm{P̂}}_{l}^{\left(\eta \right)}=\eta {\mathrm{\Pi ̂}}_{\leq l}$ , and the block from which **Ω**[**P**_*l*_] is built are unchanged. The weights depend only on the corresponding natural occupations. Thus, every term in ([Disp-formula eq15]) is fixed by the density operator itself, up to the already fixed choice of orthonormal atomic representation and atomic partition.

It remains to compare the spin-summed and per-spin descriptions of a spin-unpolarized density. Let the spin-summed density have distinct natural occupations ν_*l*_ and spectral projectors Π̂_*l*_. In the spin-summed convention, η = 2 and the weights are $\frac{1}{2}\nu_{l}$ , so

$$\Omega_{\mathrm{spin}‐\mathrm{summed}}=\underset{l}{\sum }\frac{\nu_{l}}{2}\left(\Omega \left[2{\mathrm{\Pi ̂}}_{\leq l}\right]-\Omega \left[2{\mathrm{\Pi ̂}}_{\leq l-1}\right]\right)$$

For one spin block of the same spin-unpolarized density, $P^{\varsigma }=\frac{1}{2}D$ , the projectors are the same but the natural occupations are ν_*l*_/2. The one-spin construction therefore gives

$$\Omega^{\varsigma }=\underset{l}{\sum }\frac{\nu_{l}}{2}\left(\Omega \left[{\mathrm{\Pi ̂}}_{\leq l}\right]-\Omega \left[{\mathrm{\Pi ̂}}_{\leq l-1}\right]\right)$$

The map **X** → **Ω**[**X**] is homogeneous of degree two: the coupling matrix is linear in the density block, whereas the BOO weights are squared singular values. Hence, for any non-negative scalar *a*,

$$\Omega \left[aX\right]=a^{2}\Omega \left[X\right]$$

Using this identity with *a* = 2 gives

$$\begin{array}{ll} \Omega_{\mathrm{spin}‐\mathrm{summed}} & =\underset{l}{\sum }2\nu_{l}\left(\Omega \left[{\mathrm{\Pi ̂}}_{\leq l}\right]-\Omega \left[{\mathrm{\Pi ̂}}_{\leq l-1}\right]\right),\\ \Omega_{\mathrm{per}‐\mathrm{spin}} & =2\left(\Omega^{\alpha }+\Omega^{\beta }\right)=4\Omega^{\varsigma }=\underset{l}{\sum }2\nu_{l}\left(\Omega \left[{\mathrm{\Pi ̂}}_{\leq l}\right]-\Omega \left[{\mathrm{\Pi ̂}}_{\leq l-1}\right]\right) \end{array}$$

The two operators are therefore identical. Since the spinless coupling matrix is also fixed by the same total density, the trace and the summed weight of every complete eigenspace of **M** obtained from ([Disp-formula eq12]) are identical as well; identical individual weights additionally require the same choice of basis inside any degenerate eigenspace of **M**.

Proposition 3 concerns spin-unpolarized densities. The same degree-two homogeneity has a second useful consequence for spin-pure open-shell determinants: the integer-occupation structure of the total density separates the doubly and singly occupied spaces, allowing the total-density layer construction to reproduce the per-spin Mayer operator without explicitly starting from the two spin blocks. This special determinant limit is stated separately because it is exact for ROHF-type densities but not for a spin-contaminated unrestricted density.

**Proposition 4** (Exactness for spin-pure open-shell determinants). *For a spin-pure ROHF-type determinant, the spin-summed layer construction applied to the total density*

$$D=P^{\alpha }+P^{\beta }$$

*is identical to the per-spin Mayer bond-order operator*

$$2\left(\Omega \left[P^{\alpha }\right]+\Omega \left[P^{\beta }\right]\right)$$

*Consequently the trace of the total-density layer construction is the per-spin Mayer index, evaluated without explicitly starting from the two spin blocks.*

*Proof*. Choose the spin convention so that the singly occupied orbitals belong to the α block. Let **Π**_d_ and **Π**_s_ be the orthogonal projectors onto the doubly and singly occupied spatial subspaces. Then

$$P^{\beta }=\Pi_{d},,P^{\alpha }=\Pi_{d}+\Pi_{s},,D=2\Pi_{d}+\Pi_{s}$$

The spin-summed total density therefore has natural occupations 2, 1, and 0, with spectral projectors **Π**_d_, **Π**_s_, and the virtual projector, respectively. In the spin-summed convention η = 2, so the occupation-scaled cumulative layers are

$$P_{1}=2\Pi_{d}=2P^{\beta },,P_{2}=2\left(\Pi_{d}+\Pi_{s}\right)=2P^{\alpha }$$

The corresponding layer weights are

$$w_{1}=\frac{1}{2}\left(2\right)=1,,w_{2}=\frac{1}{2}\left(1\right)=\frac{1}{2}$$

Substitution into ([Disp-formula eq15]) gives

$$\begin{array}{ll} \Omega & =\Omega \left[P_{1}\right]+\frac{1}{2}\left(\Omega \left[P_{2}\right]-\Omega \left[P_{1}\right]\right)\\ & =\frac{1}{2}\Omega \left[P_{1}\right]+\frac{1}{2}\Omega \left[P_{2}\right] \end{array}$$

Using the degree-two homogeneity **Ω**[*a*{**X**] = *a* ^2^ **Ω**[**X**] then yields

$$\begin{array}{ll} \Omega & =\frac{1}{2}\Omega \left[2P^{\beta }\right]+\frac{1}{2}\Omega \left[2P^{\alpha }\right]\\ & =2\Omega \left[P^{\beta }\right]+2\Omega \left[P^{\alpha }\right]=2\left(\Omega \left[P^{\alpha }\right]+\Omega \left[P^{\beta }\right]\right) \end{array}$$

which is exactly the open-shell per-spin operator ([Disp-formula eq10]). The trace is therefore the per-spin Mayer index ([Disp-formula eq8]). If the singly occupied orbitals are assigned to the β block instead, the same argument holds with α and β interchanged.

Propositions 3 and 4 together define the precise determinant limits of the layer construction. For spin-unpolarized densities the restricted and unrestricted descriptions are identical because they represent the same 1-RDM. For spin-pure ROHF-type open shells, the integer occupations of the total density separate the doubly and singly occupied subspaces, and the spin-summed layers reconstruct the per-spin Mayer operator algebraically. These statements do not extend to a spin-contaminated unrestricted determinant: then the α and β projectors do not generate a common integer-occupation total-density layering, and the spin-summed construction from **P** ^α^ + **P** ^β^ need not reproduce the per-spin Mayer index. The difference is the bond-order manifestation of spin contamination. The per-spin branch of Section 3 retains the determinant exchange contraction for the supplied spin blocks and therefore gives the open-shell Mayer index of that determinant; it does not purify the wave function of spin contamination, which remains a property of the supplied density.

The exact ROHF reconstruction also assumes that the analysis space resolves the doubly and singly occupied projectors. In practice this is guaranteed in the untruncated NAO representation used for the main calculations. If the density is projected onto a reduced minimal-valence space that is not invariant under the occupied projectors, the occupations are no longer exactly 2, 1, and 0, and the algebraic reconstruction can acquire a small residual.

### 4.3 Direct and Layer-Defined Indices: A Two-Orbital Example

The distinction between the two correlated extensions can be illustrated analytically. Consider two orthonormal atomic functions χ_*A*_ and χ_*B*_ and their bonding and antibonding combinations,

$$\phi_{g}=\frac{\chi_{A}+\chi_{B}}{\sqrt{2}},,\phi_{u}=\frac{\chi_{A}-\chi_{B}}{\sqrt{2}}$$

For a spin-unpolarized two-electron density, assign the natural occupations *n*_*g*_ = 1 + *x* and *n*_*u*_ = 1 – *x*, with 0 ≤ *x* ≤ 1. In the atomic basis,

23 $$D=\left(\begin{array}{ll} 1 & x\\ x & 1 \end{array}\right)$$

The atomic populations remain equal to one throughout, while the interatomic density element is *D*_*AB*_ = *x*.

For 0 < *x* < 1, the occupation-scaled cumulative layers and their gap weights are

$$\begin{array}{ll} P_{1} & =2\left|\phi_{g}\right\rangle \left\langle \phi_{g}\right|,a_{1}=x,\\ P_{2} & =2I,a_{2}=\frac{1-x}{2} \end{array}$$

They reconstruct the density exactly: **D** = *a*_1_ **P**_1_ + *a*_2_ **P**_2_. The first layer has unit atom-pair bond order, whereas the second has no interatomic block and hence zero bond order. Consequently,

24 $$\begin{array}{ll} W_{AB}^{\mathrm{dir}} & ={|D_{AB}|}^{2}=x^{2}\\ W_{AB}^{\mathrm{layer}} & =a_{1}\left(1\right)+a_{2}\left(0\right)=x \end{array}$$

These expressions extend continuously to *x* = 0 and *x* = 1. In particular, occupations 1.5 and 0.5 give $W_{AB}^{\mathrm{dir}}=0.25$ and $W_{AB}^{\mathrm{layer}}=0.50$ . Both definitions recover unit bond order for the doubly occupied bonding orbital and zero bond order at equal occupations.

For *x* > 0, both operators have the same bonding orbital but different weights:

$$\begin{array}{ll} \Omega_{\mathrm{dir}} & =x^{2}\left|\phi_{g}\right\rangle \left\langle \phi_{g}\right|,\\ \Omega_{\mathrm{layer}} & =x\left|\phi_{g}\right\rangle \left\langle \phi_{g}\right| \end{array}$$

Thus, each orbital resolution preserves the trace of its own operator; trace preservation does not identify the two correlated indices.

The additional population-normalization criterion is visible in the complete quadratic kernels. Direct evaluation and eq 18 give, respectively,

25 $$\begin{array}{ll} K^{\mathrm{dir}} & =\left(\begin{array}{ll} 1 & x^{2}\\ x^{2} & 1 \end{array}\right),\\ K^{\mathrm{layer}} & =\left(\begin{array}{ll} 2-x & x\\ x & 2-x \end{array}\right) \end{array}$$

Their row sums are 1 + *x* ^2^ and 2, respectively. The layer kernel therefore retains the prescribed marginal 2*D*_*μμ*_ = 2 at every occupation imbalance, whereas the direct kernel satisfies this identity only at the determinant limit *x* = 1. Since each atom carries one basis function in this model, the diagonal and off-diagonal entries explicitly separate the intra- and interatomic contributions to this normalized population allocation.

In this symmetric example, independently row-normalizing either kernel would produce a Markov matrix with a uniform stationary distribution. The distinguishing criterion is therefore not the mere possibility of constructing a stationary process, but retention of the projector-derived normalization *K*_*μν*_/(2*D*_*μμ*_) together with the physical population marginals. The linear dependence on *x* in eq 24 illustrates the consequence of that choice, rather than a claim that the larger value is closer to an exact two-particle delocalization index or to an energetic measure of bond strength.

## 5 Optional Symmetry Labeling

The bond-order orbitals and their weights are defined independently of any symmetry analysis; the unified index of Sections 2–1 stands on its own. The labeling here is an optional readout that analyses the angular content of each BOO with respect to the bond axis **n̂** = (**R**_*B*_ – **R**_*A*_)/|**R**_*B*_ – **R**_*A*_|, on which both atomic centers lie. A BOO is in general not an eigenfunction of the projected angular momentum **n̂** · **L**; its weight is instead resolved into the eigenspaces |*m*′| = 0, 1, 2, 3 of that operator, which correspond to σ, π, δ, φ character. For each (atom,n,l) shell we form (**n̂** · **L**)^2^ in the real-spherical-harmonic basis of degree l (via the standard angular-momentum matrices and the real ↔ complex transform^60^), a real symmetric PSD matrix with eigenvalues *m* ^′2^; projecting the BOO’s per-shell coefficients onto its eigenspaces and bucketing the weight by |*m*′| yields the σ/π/δ/φ content. A BOO is labeled by the dominant channel when its fractional weight exceeds a purity threshold. Being a genuine rotation, the labeling is valid for an arbitrary bond direction and for any l≤4 , i.e., also for *d*- and *f*-block bonds. Invariance of the operator trace and of the summed weight of a complete degenerate subspace does not imply invariance of channel totals obtained by assigning individual vectors to their dominant angular label; within a degenerate subspace the individual labels, and hence the channel totals, can depend on the basis chosen inside that subspace.

## 6 Computational Details

Benzene densities were obtained with Gaussian 16 ^61^ using def2-QZVPP (equivalent to dhf-QZVPP for C and H^62^): RHF, UHF, RMP2 and UMP2 for the singlet, and UHF, ROHF and UMP2 for the triplet. The main-group and transition-metal clusters were treated at PBEh-3c,^63^ whereas the actinide examples used PBE0-D4/SARC-DKH-TZVP with second-order Douglas–Kroll–Hess relativity.^64,65^ The ${\mathrm{Th}}_{3}^{q}$ series used the fixed experimental ring geometry,^66^ and U_2_ was evaluated at its literature bond length.^67^ Cluster calculations used ORCA 6.1.1 ^68,69^ with RIJCOSX.^70^ Detailed geometries and electronic-structure settings are given in the Supporting Information (SI).

Bond-order analyses used the Natural Bond-Order Analysis (NBA) module of runEDDB (build of 27 September 2026),^5,71,72^ with the full natural atomic orbital (NAO) basis and the rigorous NBO-type construction. Determinant densities use the expressions of Sections 2–3; correlated densities use the spectral-layer extension of Section 4. Both the grouping-gap and group-discard thresholds were 10^–6^. All reported indices and channel sums include the complete coupling frame before filtering for display or export. Contributions not assigned by the angular classifier are retained as “other”. The implementation, pair-selection and export thresholds, and numerical checks are documented in the SI.

The relaxed MP2 densities contained natural occupations outside the admissible range. Before analysis, each density channel was therefore subjected to an electron-number-preserving spectral projection into [0, η] (−force-nrep), leaving its natural orbitals unchanged. This preprocessing is separate from the bond-order definition and need not preserve the original atomic populations. Per-spin and total-density analyses of the triplet use the same spin blocks, spectrally corrected for MP2; total-density entries were evaluated with an auxiliary Python script in the same NAO basis. The projection formula, its numerical effect, residual QD errors and script validation are provided in the SI.

## 7 Results and Discussion

The examples below are not intended as a benchmark of electronic-structure methods. They are diagnostic tests of the bond-order-orbital construction. Each system was chosen because it stresses a different part of the formalism: restricted–unrestricted invariance, open-shell spin resolution, conflicting σ/π aromaticity, the *d*/*f*-block σ/π/δ hierarchy, and incipient multicenter actinide delocalization.

### 7.1 Invariance and Spin Resolution: Benzene

Benzene tests the consistency of the construction while retaining a bond-resolved interpretation. Table 1 reports the sum over the six ring C–C bonds in the untruncated NAO basis. The HF entries use the determinant bond-order expressions, whereas the MP2 entries use the spectral-layer extension applied to the spectrally corrected relaxed MP2 densities (Section 6), not the direct quadratic contraction of the correlated density (which would give 7.994 for the singlet). For the singlet, restricted and unrestricted evaluations agree to the printed precision within each level of theory (the RMP2 and UMP2 sums differ by 3 × 10^–5^, and no per-bond channel value differs after rounding to three decimals). This agreement illustrates Proposition 3: equivalent spin bookkeeping of the same spin-unpolarized density gives the same layer-defined operator. It does not imply equality between the direct and layer-defined correlated indices.

**Table 1:** Ring-Summed C–C Bond Indices and Channel Contributions for Benzene (Def2-QZVPP, Full NAO Basis)^a^

Wave function	Treatment	Σ*W*	Σσ	Σπ	Σrest
*Ground-state singlet* S_0_					
RHF = UHF	either	8.642	5.865	2.777	0.000
RMP2 = UMP2	either	8.362	5.702	2.643	0.017
*Lowest triplet* T_1_					
UHF	per-spin	7.660	5.875	1.784	0.001
	total-density	7.797	5.879	1.918	0.001
ROHF	per-spin	7.944	5.881	2.063	0.000
	total-density	7.944	5.881	2.063	0.000
UMP2	per-spin	7.780	5.711	2.040	0.029
	total-density	7.794	5.711	2.054	0.029

a MP2 entries use spectrally corrected relaxed densities; the triplet treatments start from the same spin blocks (see text). Rest = δ + φ + other. Restricted and unrestricted singlet values agree to the reported precision. Full channel sums are given in the SI.

For the triplet, the table compares analysis of the separate spin densities with spectral-layer analysis of the total density. The per-spin route uses eq 8 for UHF and ROHF; for UMP2, the layer construction is applied separately to the two correlated spin blocks before their operators are combined. The total-density route instead applies the η = 2 layer construction to **D** = **P** ^α^ + **P** ^β^. It should not be confused either with direct evaluation of ${∥D_{AB}∥}_{F}^{2}$ or with the subsequent display of a per-spin operator in a common spinless orbital frame.

The two routes coincide for ROHF because the integer occupation structure of its total density permits the exact reconstruction established in Proposition 4. That equivalence is not guaranteed for a general UHF density or for the correlated UMP2 density. The differences reported in Table 1 therefore characterize the dependence of the layer-defined index on retaining or combining the spin information; they are not, by themselves, quantitative measures of spin contamination. Likewise, changes between HF and MP2 entries describe changes in the chosen one-particle covalency descriptor, not changes in an energetic bond-strength measure. The spectral correction of the MP2 densities changes the triplet sums by −0.004 (Σ*W*, per-spin) and −0.012 (total-density) relative to the same analysis of the uncorrected densities, and the singlet sums by less than 10^–5^; the uncorrected values are given in the Supporting Information.

### 7.2 Channel Competition in Square and Rectangular Al_4_ Rings

After benzene has tested the formal invariance of the construction, the ${\mathrm{Al}}_{4}^{2-}/{\mathrm{Al}}_{4}^{4-}$ pair shows why resolving a scalar bond order into orbital channels is chemically useful. These two all-metal rings are established examples of contrasting aromatic behavior: the square ${\mathrm{Al}}_{4}^{2-}$ core is assigned double σ + π aromaticity, whereas the rectangular ${\mathrm{Al}}_{4}^{4-}$ core combines a delocalized σ framework with an antiaromatic, symmetry-broken π system.^73–75^ They are represented here by the corresponding Li_2_Al_4_ and Li_4_Al_4_ clusters; only the Al–Al entries are discussed, because the Li atoms act as spectator counterions for the present channel comparison.

The BOO decomposition is not, by itself, an aromaticity index: its channel weights are non-negative and therefore do not assign an aromatic or antiaromatic sign. What it does reveal directly is whether the covalency in a given symmetry channel remains distributed uniformly around the ring or becomes localized into alternating bonds. Table 2 therefore provides an orbital-resolved bonding counterpart to the established aromaticity assignments.

**Table 2:** Bond Indices and *σ*/*π* Contributions for the Al_4_ Cores in Li_2_Al_4_ and Li_4_Al_4_ ^a^

Bond	*W*_*AB*_	σ	π
*Square* ${\mathrm{Al}}_{4}^{2-}$ *core*			
edge (×4)	1.052	0.710	0.342
diagonal (×2)	0.773	0.500	0.274
*Rectangular* ${\mathrm{Al}}_{4}^{4-}$ *core*			
short edge (×2)	1.734	0.730	1.004
long edge (×2)	0.949	0.803	0.145
diagonal (×2)	0.565	0.507	0.058

a PBEh-3c densities. Values are averaged over the indicated contacts; multiplicities appear in parentheses. Weights outside the displayed channels are below 0.0005 per contact and are not added to σ. Full pair data are given in the SI.

In square ${\mathrm{Al}}_{4}^{2-}$ , all four edges carry the same σ (0.710) and π (0.342) contributions. The cross-ring diagonals retain substantial weights in both channels, including a π component of 0.274, consistent with covalency distributed over the whole four-membered ring rather than confined to its edges. The decomposition therefore exposes the simultaneous cyclic delocalization of the σ and π frameworks associated with the established double-aromatic description.

Adding two electrons changes primarily the π channel. In rectangular ${\mathrm{Al}}_{4}^{4-}$ , the σ contributions remain comparatively uniform on the short and long edges (0.730 and 0.803, respectively), whereas the π contribution changes from 1.004 on a short edge to only 0.145 on a long edge. This nearly 7-fold alternation identifies the π system as the source of the rectangular bond localization. The scalar bond orders already show that the edges are inequivalent, but only the resolved analysis shows that the distortion is not produced by the σ framework: it is overwhelmingly a π-channel effect. This selective localization is the bond-order counterpart of the established σ-aromatic/π-antiaromatic description.

Two reference rings provide compact checks on this interpretation at the same level of theory. Square ${\mathrm{Ge}}_{4}^{2+}$ , isoelectronic with ${\mathrm{Al}}_{4}^{2-}$ , again shows uniform edge contributions (*W*_*AB*_ = 1.050, σ = 0.618, π = 0.432), whereas the six-π-electron cyclo- $P_{5}^{-}$ ring gives uniform values of *W*_*AB*_ = 1.410, σ = 0.938, and π = 0.472. Their role is not to establish a separate aromaticity scale, but to verify that the channel classifier recovers the expected cyclic π delocalization in chemically distinct main-group rings.

### 7.3 The *d*/*f*-Channel Hierarchy and Actinide σ Delocalization

Bonds and rings built from *d* and *f* orbitals add channels that no *p*-block ring can show. Table 3 collects the systems that test this extended channel alphabet: a triply aromatic *d*-metal ring, an open-shell congener treated through the spinless projection scheme, a canonical metal–metal δ bond, a heavy-element multiple bond, and an actinide charge series in which a nominally two-center Th–Th edge becomes a window on three-center σ delocalization. The values are per symmetry-distinct contact; within rounding, *W*_*AB*_ = σ + π + δ + other, where “other” is the weight that the optional two-center labeling does not assign to a single channel. No single φ-labeled component reaches the export cutoff in any of these single-determinant densities.

**Table 3:** Bond Indices and Angular-Channel Contributions for Selected *D*- and *F*-Block Systems^a^

System	Bond or contact	*n*_*e*_	*W*_*AB*_	σ	π	δ	Other
*Metal–metal and d*/*f*-*block channel hierarchy*							
${\mathrm{Hf}}_{3}{(}^{1}A_{1}^{\prime })$	Hf–Hf (×3)	–	2.072	0.739	0.889	0.444	0.000
${\mathrm{Ta}}_{3}^{-}\left(\mathrm{quintet}\right)$	Ta1–Ta2, Ta2–Ta3 (×2)	–	2.168	0.818	0.965	0.169	0.216
	Ta1–Ta3 (×1)	–	2.157	0.838	0.974	0.276	0.068
Re2Cl82−	Re–Re	–	2.562	0.754	1.267	0.541	0.000
U_2_ (septet)	U–U	–	3.257	1.120	2.001	0.136	0.000
${\mathrm{Th}}_{3}^{q}$ *charge series at fixed ring geometry*							
${\mathrm{Th}}_{3}^{10+}$	Th–Th (×3)	2	0.447	0.002	0.001	0.000	0.444
${\mathrm{Th}}_{3}^{4+}$	Th–Th (×3)	8	1.506	0.937	0.507	0.000	0.062
Th_3_	class A: Th1–Th2, Th1–Th3 (×2)	12	1.547	0.583	0.951	0.013	0.001
Th_3_	class B: Th2–Th3 (×1)	12	2.124	0.556	1.391	0.177	0.001

a Values are averaged over the indicated contacts; multiplicities appear in parentheses. *n*_*e*_ counts ring valence electrons in ${\mathrm{Th}}_{3}^{q}$ . Other denotes unassigned weight; each φ channel sum is below 10^–6^. Methods and individual pair data are given in the SI.

The first block of Table 3 demonstrates the ordinary σ/π/δ hierarchy. The triangular Hf_3_ cluster, in its A1′1 ground state, is the textbook triply (σ + π + δ) aromatic ring:^76^ each equivalent edge resolves into balanced σ (0.739), π (0.889), and δ (0.444) components. Its open-shell congener ${\mathrm{Ta}}_{3}^{-}$ has two contacts near 2.450 Å and a third at 2.465 Å, listed separately in the table. It carries the same three channels through the spinless projection scheme of Section 3, but with a weaker δ component (0.169 on the two close contacts and 0.276 on the third), consistent with a partially occupied *d*_δ_ manifold. In this spin-polarized *d* manifold a further 0.07–0.22 per contact is carried by coupling-frame vectors of mixed angular character that the 60% purity criterion does not assign to a single channel; this weight is reported in the “other” column rather than being attributed to σ. The Re–Re bond in Re2Cl82− gives the canonical metal–metal δ bond: the index separates into one σ, two π, and one δ contribution, the orbital-resolved σ^2^π^4^δ^2^ quadruple bond of Cotton.^77^

The U_2_ entry is a useful negative control. Although the multireference quintuple-bond picture of diuranium famously involves 5*f* orbitals,^67^ the present single-determinant density resolves the U–U index into σ + 2π + δ, and no individual φ-labeled component reaches the reporting threshold (the φ channel sum is below 10^–6^). The classifier therefore does not manufacture a high-angular-momentum bond merely because *f* functions are available: it reports φ weight only when the bond-order density actually carries such overlap. A larger φ contribution, if present in the supplied 1-RDM, would be detected by the same angular-momentum analysis; this statement concerns the bond-order density of the single-determinant U_2_ density used here and is not a separate proof that all 5*f*_φ_ electrons are nonbonding.

The second block of Table 3 follows the ${\mathrm{Th}}_{3}^{q}$ charge series at the fixed experimental ring geometry.^66^ In ${\mathrm{Th}}_{3}^{10+}$ , where only two electrons occupy the ring, the Th–Th edge index is small (0.447) and consists almost entirely of one component (0.444) that the two-center classifier does not assign to any channel (σ = 0.002, π = 0.001); it appears in the “other” column. The classifier result and the chemical interpretation must be kept apart. “Other” records only that the angular content of this component with respect to a single Th–Th axis is not dominated by one |*m*′| value; it does not by itself indicate a nonbonding or Rydberg contribution, nor the absence of σ delocalization; the absence of a two-center label does not by itself establish multicenter character either. The result is, however, consistent with the 3c–2e interpretation of the two-electron ring,^66^ in which the component is the two-center projection of a ring orbital whose σ character is defined with respect to the ring rather than to one edge, so that the two-center analysis is flagging an object that the multicenter construction of Section 8 represents directly. Filling the ring buries this limiting picture. At 4+ the index is a more conventional σ + π framework (σ = 0.937, π = 0.507, with 0.062 unassigned), and neutral Th_3_ develops appreciable σ + π + δ bonding while electronically separating the three identical Th–Th contacts into two classes, class A (Th1–Th2, Th1–Th3) and class B (Th2–Th3), at the unchanged Th–Th distance of 3.989 Å. The *pair* BOO analysis thus reveals the natural entry point to the multicenter construction of Section 8: the two-center operator detects the projection of a collective three-center mode, whereas the multicenter operator represents that mode directly.

## 8 Natural Multicenter Extension

The two-center construction is the smallest member of a more general operator family. Once the molecular one-particle space has been partitioned into mutually orthogonal atomic subspaces, replacing an atom pair by a set of atoms requires no change in the underlying logic. Let

$$C=\left\{A_{1},...,A_{m}\right\}$$

be such a set, and let **X** denote a spin-summed density, a one-spin density, or an occupation-scaled density layer of Section 4. The multicenter coupling operator is obtained by retaining every interatomic block inside C and removing the one-center blocks:

26 $${\left(M_{C}\left[X\right]\right)}_{AB}=\left\{ \begin{array}{ll} 0, & A=B,\\ X_{AB}, & A\neq B, \end{array}\right.,A,B\in C$$

For C={A,B} , eq 26 reduces exactly to the two-center coupling matrix ([Disp-formula eq4]). For *m*  > 2, its eigenvectors are collective coupling modes with amplitudes distributed over any number of the participating atomic subspaces.

The positive spectral branch defines the corresponding multicenter bond-order density and its trace:

27 $$\Omega_{C}\left[X\right]={[M_{C}[X]]}_{+}^{2}=\underset{\lambda_{r}>0}{\sum }\lambda_{r}^{2}u_{r}u_{r}^{T},,W_{C}\left[X\right]=\mathrm{Tr}\Omega_{C}\left[X\right]=\underset{\lambda_{r}>0}{\sum }\lambda_{r}^{2}$$

where $M_{C}u_{r}=\lambda_{r}u_{r}$ and [**M**]_+_ denotes the positive spectral part of a Hermitian matrix. The vectors **u**_*r*_ with λ_*r*_ > 0 are therefore the natural multicenter counterparts of the two-center BOOs, and their non-negative weights sum exactly to the multicenter index $W_{C}$ .

This extension inherits the central algebraic properties of the pair theory. The operator $\Omega_{C}$ is positive semidefinite, invariant under unitary rotations within each fixed atomic subspace, and homogeneous of degree two in the density:

$$\Omega_{C}\left[aX\right]=a^{2}\Omega_{C}\left[X\right]$$

Consequently, no separate open-shell or post-Hartree–Fock prescription is required. For an open-shell determinant one forms $2\left(\Omega_{C}\left[P^{\alpha }\right]+\Omega_{C}\left[P^{\beta }\right]\right)$ ; for a correlated density one replaces the pair operator **Ω**[**P**_*l*_] in eq 15 by $\Omega_{C}\left[P_{l}\right]$ . The same per-spin assembly, occupation-scaled spectral layers, non-negativity argument, and exact single-determinant limits therefore carry over directly. In the two-center limit,

$$\Omega_{\{A,B\}}=\Omega_{AB},,W_{\{A,B\}}=W_{AB}$$

The existence of this multicenter extension does not make the resolved two-center description a lower-level approximation. The two constructions answer different questions. A multicenter scalar compresses the collective coupling of an entire atomic set, whereas the collection of pair-resolved symmetry-channel indices retains the spatial structure of that coupling: which bonds carry it, which symmetry channel transmits it, and where localization or bond alternation occurs. Established multicenter indices and EDDB-type descriptors provide valuable global measures of cyclic delocalization,^4,5,71^ but no single cluster number can retain the complete bond-by-bond and channel-by-channel pattern from which it is condensed. Comparisons between rings containing different numbers of centers additionally require an explicit choice of normalization, whereas every *W*_*AB*_ retains the same local meaning.

The ${\mathrm{Al}}_{4}^{2-}/{\mathrm{Al}}_{4}^{4-}$ comparison of Section 7.2 (Table 2) illustrates the distinction: the pair-resolved analysis identifies both the channel responsible for the symmetry breaking of the rectangular core and the edges on which the π covalency becomes localized. A global multicenter index can summarize the cyclic cooperation, but it cannot replace this more highly resolved description.

The pair and multicenter operators are thus complementary rather than hierarchical. The multicenter trace $W_{C}$ is available when a global measure of collective coupling is desired; the set of resolved pair indices may already provide the more complete diagnostic of resonance structure, bond equalization, or symmetry-selective localization. The aim is not to force every bonding problem into one number, but to retain exactly as much of the density-matrix information as the chemical question requires.

## 9 Conclusions

Bond order is useful because it condenses electronic structure into a chemically interpretable quantity. An operator resolution retains that simplicity while exposing the orbital contributions hidden by the scalar index. Within a fixed orthonormal atomic partition, the present framework defines bond indices as traces of positive semidefinite atom-pair operators. It recovers the closed-shell Wiberg and open-shell per-spin Mayer forms in their respective determinant limits; spin-pure ROHF-type densities also permit reconstruction of the per-spin operator from the integer occupation layers of the total density.

For correlated densities, the cumulative spectral-layer construction defines a particular continuation of this projector-based analysis, not merely a different orbital resolution of the directly evaluated quadratic index. Its additional criterion is population consistency. For each density channel, the exact occupation-gap weights reconstruct the supplied 1-RDM, while the layer-weighted quadratic population kernel retains its orbital and atomic population marginals. Their normalized distribution is stationary under the associated Markov transition matrix without requiring idempotency of the physical density. Positivity and recovery of the determinant limits are retained, but these properties alone do not distinguish the layer construction from direct quadratic evaluation. Nor does stationarity uniquely determine a correlated bond order or replace the full exchange–correlation information contained in the two-particle density.

The two-orbital example makes this distinction explicit: direct and layer-defined operators can have the same bonding orbital but different weights at fractional occupations. Each orbital resolution preserves the trace of its own operator, not a common correlated index shared by both definitions. The exact layer construction is continuous with respect to the supplied density in a fixed finite-dimensional atomic representation and requires no near-degeneracy threshold. Replacing nearby occupations by group means is a separate numerical approximation, whose effects must not be confused with the invariance of the exact construction under rotations within occupation-degenerate eigenspaces.

The choice of orbital representation is likewise distinct from the definition of the bond index. Separate spin-resolved BOOs retain explicit spin information, whereas diagonalization of the combined bond-order operator gives its exact orthonormal spectral resolution. Projection onto a complete common spinless basis instead reports the spin-resolved weights in the spatial coupling modes of the total density. This display preserves the total index but generally does not reconstruct the full operator from its diagonal weights alone. The three representations are therefore complementary; the common spinless frame is a practical reporting choice, not a uniquely privileged description.

The molecular examples demonstrate the diagnostic value of retaining both bond and channel resolution. Benzene illustrates the agreement of restricted and unrestricted descriptions of the same spin-unpolarized density and the role of spin resolution in open-shell analysis. The ${\mathrm{Al}}_{4}^{2-}/{\mathrm{Al}}_{4}^{4-}$ comparison separates comparatively uniform σ covalency from pronounced π-bond alternation, identifying both the affected symmetry channel and the edges on which localization occurs. The transition-metal and actinide examples extend the analysis to σ, π, and δ contributions; no φ component is reported above threshold for the tested densities. These results characterize the supplied electronic structures rather than establishing a universal aromaticity scale or a measure of energetic bond strength.

The multicenter extension of Section 8 applies the same operator construction and per-spin and spectral-layer assembly to larger atomic sets. Its trace is a potentially useful collective diagnostic, but greater compression does not necessarily provide greater chemical insight. A single cluster value cannot retain the distribution of covalency over individual bonds and symmetry channels, and comparisons between atomic sets of different sizes require attention to normalization. Pair-resolved indices retain the same local definition within the chosen atomic partition and can therefore provide the more informative account of bond alternation and symmetry-selective localization.

Implemented in runEDDB, the framework provides a common operator language for determinant and correlated-density analyses while making the choices behind the correlated extension explicit. Its purpose is not to select one universal number for every bonding problem, but to connect a specified population-allocation criterion with an orbital-resolved description of chemical covalency.

## Supplementary Material



## Data Availability

**Data and Software Availability:** The numerical data underlying the reported tables are contained in the article and the Supporting Information. The Supporting Information provides detailed computational protocols, molecular geometries, representative input files, runEDDB analysis settings, annotated program output, the per-pair channel resolution of every reported bond, and the diagnostics of the spectral correction applied to the MP2 densities; the accompanying numerical material contains the runEDDB commands, logs, and full-precision diagnostic files of every analysis, and the auxiliary Python scripts used for the total-density construction and for the independent checks. The runEDDB release implementing the quadratic bond-order analysis described in this work is freely available from the runEDDB website: https://aromaticity.uj.edu.pl/runeddb/download.html.

## Notes

It contains the detailed computational protocol and thresholds; an annotated example of the runEDDB bond-order-orbital output; the per-pair channel resolution of every reported bond; the diagnostics of the spectral correction applied to the MP2 densities and a comparison with the uncorrected analysis; the complete channel resolution of the benzene ring sums; a description and validation of the auxiliary Python evaluation; an analytic three-function comparison of the spin-resolved, spectral, and coupling-frame representations; Cartesian geometries and input fragments for the reported calculations; and information on the computing resources used (PDF)
