# Supplementary material for: A Unified Operator Resolution of Quadratic Bond Orders
Source: J Phys Chem A. 2026 Oct 10;130(42):8529–43. doi: 10.1021/acs.jpca.6c04675 (PMC13700101; doi:10.1021/acs.jpca.6c04675)
Supplement: Supplementary file 1 [file jp6c04675_si_001.pdf]

Supporting Information

A Unified Operator Resolution of Quadratic Bond Orders

Dariusz W. Szczepanik

K. Gumiński Department of Theoretical Chemistry, Faculty of Chemistry, Jagiellonian University, Gronostajowa 2, 30-387 Kraków, Poland.
E-mail: dariusz.szczepanik@uj.edu.pl

Table of contents

S1 Computational details S2
S2 Example runEDDB output S4
S3 Per-pair channel resolution for the cluster set S4
S4 Spectral correction of the MP2 densities and its residuals S5
S5 Full channel resolution of the benzene ring sums S8
S6 Auxiliary Python evaluation of the total-density construction S8
S7 Analytic comparison of the three orbital representations S9
S8 Input files and geometries used S10
S8.1 All-metal and inorganic clusters (PBEh-3c) S10
S8.2 Actinide systems (PBE0/SARC-DKH-TZVP, DKH2) S12
S9 HPC resources S12

S1 Computational details

The quadratic bond-order analysis and its bond-order-orbital (BOO) resolution are implemented in the Natural Bond-order Analysis (NBA) module of the runEDDB program. The program constructs the natural atomic orbital (NAO) representation internally and reads density matrices from formatted-checkpoint, Molden, ORCA, and other standard wavefunction files. All runEDDB calculations reported in the main text and in this document used the same executable (build of 27 September 2026, SHA-256 `c6b58b0a...e1035`), the untruncated NAO basis (`-b nao`) constructed with the rigorous NBO-type algorithm (`--nao-algorithm rigorous`), and the same protocol. Within this fixed atomic partition, determinant densities recover the closed-shell Wiberg or open-shell per-spin Mayer form, whereas correlated densities are analysed with the spectral-layer extension of Section 4 of the main text. The only results not produced by runEDDB itself are the total-density entries for the spin-resolved triplet densities, which were evaluated with the auxiliary Python script of Section S6 in the same NAO representation and with the same thresholds; they are marked as such wherever they appear. Every runEDDB job was run with the same command pattern,

```
--global -b nao --nao-algorithm rigorous --wbo-thresh 0.001 -n 16 -v
        --table no,bo,ao,npa --export-fchk no,ao,bo
```

plus `--density-source MP2` for the Gaussian MP2 checkpoints and `--force-nrep` for the MP2 densities of the main tables; the exact commands, logs, and diagnostic files are part of the numerical material accompanying this Supporting Information.

Implementation and orbital export. For each selected atom pair, NBA diagonalizes the total-density coupling matrix $\mathbf{M}$ to obtain a complete orthonormal frame $\mathbf{Q}$. The closed-shell, per-spin, or spectral-layer operator $\boldsymbol{\Omega}$ is then constructed as defined in Sections 2–4 of the main text, and the projected weights $n_k = \langle \mathbf{m}_k | \boldsymbol{\Omega} | \mathbf{m}_k \rangle$ are evaluated for every column of $\mathbf{Q}$. Positive, negative, and null branches of $\mathbf{M}$ are all retained. The calculation requires symmetric eigenproblems of dimension $n_A + n_B$, including separate spin and layer problems where applicable; angular labeling uses per-shell problems of dimension $2\ell + 1 \leq 9$. Selected atom-pair modes are back-transformed to the original AO basis and exported with their projected weights stored as orbital occupations.

Three thresholds enter the analysis and are kept distinct. (i) Layer grouping: the natural occupations are sorted in decreasing order and a new quasi-degenerate group is started whenever two consecutive occupations differ by 10^{-6} or more (gap threshold); a completed group whose mean occupation is below 10^{-6} (drop threshold) is discarded. Both values are the program defaults and were not modified. (ii) Pair gates: the connectivity mask uses a gate of 10^{-3} on the internal gating index of runEDDB, which is one half of the closed-shell Wiberg index (the sum of the two one-spin contractions for open shells); the bond-orbital analysis uses ten times this value, 10^{-2}, which corresponds to $W_{AB} \geq 0.02$ in the convention of the main text. No pair of the reported tables lies close to this gate. (iii) Export cutoff: components with weight below 10^{-4} are neither listed in the console table (which shows at most twelve components per pair) nor exported. All indices and channel sums reported here were taken from the full-precision diagnostic files written under `-v` (`*.NBA_DIAG.csv`, one row per gated pair; `*.NBA_MODES.csv`, one row per coupling-frame vector), which contain the complete trace, the projection sum, an independent scalar index, and the channel and branch sums evaluated over the complete coupling frame before any cutoff. For every pair the identities $\mathrm{Tr}\,\boldsymbol{\Omega} = \sum_k n_k$ (all frame vectors), $\mathrm{Tr}\,\boldsymbol{\Omega} = \sigma + \pi + \delta + \varphi + \mathrm{other}$, and $\mathrm{Tr}\,\boldsymbol{\Omega} = (\text{exported}) + (\text{omitted})$ hold to

better than 10^{-14}; for determinant densities $\mathrm{Tr}\,\boldsymbol{\Omega}$ equals the direct contraction of the density block, and for correlated densities it equals the independent scalar layer sum over the same layers (not the direct square of the correlated density). The "other" entry is the weight of frame vectors whose dominant $|m'|$ channel does not reach the 60 % purity threshold of the optional labeling; it is never added to σ.

Benzene invariance tests. The ground-state singlet (S_0, D_{6h}) and lowest triplet (T_1, D_{2h}) benzene calculations used Gaussian 16 (rev. C.01) with the def2-QZVPP basis. The closed-shell singlet was evaluated at the RHF, UHF, RMP2, and UMP2 levels, using the same RI-MP2-optimized D_{6h} geometry for the single-point calculations. The lowest triplet was evaluated at the UHF, ROHF, and UMP2 levels. The correlated entries use relaxed MP2 one-particle densities (`Density=MP2`), read from the `Total MP2 Density` and `Spin MP2 Density` blocks of the checkpoint files (never from the SCF density blocks stored in the same files). For the open-shell analyses, the spin-resolved α and β density blocks were used for the per-spin construction, whereas the total-density entries apply the $\eta = 2$ layer construction to $\mathbf{P}^{\alpha} + \mathbf{P}^{\beta}$ as a single spin-summed density. The MP2 densities of the main table were preprocessed by the spectral correction of Eq. (S1) (Section S4); the same densities analysed without this correction are reported in Section S4 for comparison. The total-density construction for spin-resolved input is not a program option; it was evaluated with the auxiliary Python script described in Section S6. The restricted/unrestricted equality for the closed-shell state was also reproduced in ORCA using the dhf-QZVPP basis; the ORCA RI-MP2 controls (Section S4) are spinless densities from an RHF and a UHF reference, respectively, and do not test explicit correlated α/β blocks.

Main-group and transition-metal clusters. The Li_2Al_4, Li_4Al_4, Ge_4^{2+}, cyclo-P_5^-, $Re_2Cl_8^{2-}$, Hf_3, and Ta_3^- systems were optimized and their densities obtained at the PBEh-3c composite level, i.e., the modified PBE hybrid with the def2-mSVP basis and D3(BJ)+gCP corrections. Closed-shell singlets were treated spin-restricted, whereas the Ta_3^- quintet was treated spin-unrestricted. These calculations provide compact and stable single-reference densities for the channel-resolution tests in the main text.

Actinide systems. The Th_3^q charge series and U_2 were treated with PBE0-D4 and the all-electron scalar-relativistic SARC-DKH-TZVP basis under the second-order Douglas–Kroll–Hess Hamiltonian. For the Th_3^q series, single-point densities were evaluated at the fixed experimental tri-thorium ring geometry, so that only the electron count changes along the charge series. The U_2 septet was evaluated at the literature U–U bond length of 2.43 Å. All cluster calculations used ORCA 6.1.1 with the RIJCOSX approximation and the def2/J or SARC/J Coulomb-fitting basis as appropriate.

The $\sigma/\pi/\delta/\phi$ decomposition reported in the main text should be read as a qualitative channel diagnostic for the stated one-particle densities, not as a benchmark of density functionals or basis sets. The exact trace relation is enforced by the BOO construction itself, whereas the chemical assignment depends on the quality and stability of the underlying density. The compact PBEh-3c basis is useful in this context because it avoids the Rydberg-driven inflation that can affect Mayer-type indices in very diffuse basis sets, while the relativistic PBE0-D4/SARC-DKH treatment provides a consistent scalar-relativistic description of the actinide examples.

S2 Example runEDDB output

The Natural Bond-order Analysis is printed by runEDDB in self-describing blocks. A representative bond-order-orbital output is shown below.

Bond-order orbitals (`--table bo`). The genuine console output for the diuranium molecule U_2 (PBE0-D4/SARC-DKH-TZVP, septet, per-spin operator $2(\mathbf{\Omega}^\alpha + \mathbf{\Omega}^\beta)$ projected onto the coupling frame of the total density), reproduced verbatim from the run used for Table 3 of the main text (the metal–metal table). Three quantities must be distinguished. The *Total* column is the complete trace $\mathrm{Tr}\,\mathbf{\Omega} = 3.25675$, i.e. the sum of the projected weights of all 318 coupling-frame vectors of the U–U pair (positive, negative, and null branches) before any cutoff. The sum of the 27 weights above the export cutoff of 10^{-4} is 3.25616 (full precision; these are the components written to the exported orbital file), the remaining 0.00059 being carried by components below the cutoff. The console shows at most twelve components per pair; the twelve values printed below add up to 3.229 when the printed five-decimal numbers are summed (3.22903 at full precision). Two of the listed σ components (0.02255 and 0.01964) belong to the negative branch of the coupling matrix $\mathbf{M}$; their weights $n_k = \langle \mathbf{m}_k | \mathbf{\Omega} | \mathbf{m}_k \rangle$ are non-negative like all others, and a negative coupling eigenvalue is not a negative bond-order contribution. The symmetry sums over the complete frame are $\sigma = 1.11974$, $\pi = 2.00135$, $\delta = 0.13566$, $\varphi < 10^{-6}$. Note the two π and the σ components dominating the U–U multiple bond; no individual φ-labelled component reaches the reporting threshold, which is a statement about the bond-order density of this single-determinant density, not an exact zero of the channel nor a separate proof that all $5f_\varphi$ electrons are non-bonding. The box-drawing frame of the console is reproduced with ASCII characters; the numbers are unchanged.

```
+--------------------------------------------------------------------------------+
|                          BOND-ORDER ORBITALS (BOO) IN NAO BASIS                |
+--------------------------------------------------------------------------------+
   Index      Atom pair          Total        BOO occupations with symmetry labels
  ------- -----------------   ---------   --------------------------------------------
       1       1~2       U~U     3.25675     0.99751(π) 0.99729(π) 0.73441(σ) 0.27112(σ)
                                             0.03623(σ) 0.03440(δ) 0.03438(δ) 0.03344(δ)
                                             0.03342(δ) 0.02255(σ) 0.01964(σ) 0.01465(σ)
  ------- -----------------   ---------   --------------------------------------------
```

S3 Per-pair channel resolution for the cluster set

Table S1 lists, for every atom pair that enters a table or a value quoted in the main text, the full bond index $W_{AB} = \mathrm{Tr}\,\mathbf{\Omega}_{AB}$ and its resolution into the σ, π, δ, and φ channels and the unassigned remainder ("other"), all evaluated over the complete coupling frame before the 10^{-4} export cutoff (runEDDB diagnostic files `*.NBA_DIAG.csv`). The distance R is taken from the geometry read by the program. Values in the main text are averages over the symmetry-equivalent contacts of this table. The φ channel sum is below 10^{-6} for every pair, and no individual φ-labelled component reaches the 10^{-4} reporting threshold in any system.

Table S1. Per-pair bond indices and channel sums for the cluster set (full NAO, rigorous NAO algorithm; PBEh-3c for the main-group and $5d$ systems, PBE0-D4/SARC-DKH-TZVP DKH2 for U_2 and Th_3^q). $W = \sigma + \pi + \delta + \varphi + \mathrm{other}$ before rounding.

System	Pair	R/Å	W_{AB}	σ	π	δ	φ	other
Li_2Al_4	Al1–Al2	2.588	1.052	0.710	0.342	0.000	0.000	0.000
	Al1–Al3	3.659	0.773	0.500	0.274	0.000	0.000	0.000

System	Pair	R/Å	W_{AB}	σ	π	δ	φ	other
	Al1–Al4	2.588	1.052	0.710	0.343	0.000	0.000	0.000
	Al2–Al3	2.588	1.052	0.710	0.342	0.000	0.000	0.000
	Al2–Al4	3.659	0.773	0.500	0.274	0.000	0.000	0.000
	Al3–Al4	2.587	1.053	0.710	0.343	0.000	0.000	0.000
Li_4Al_4	Al1–Al2	2.583	0.949	0.803	0.145	0.000	0.000	0.000
	Al1–Al3	3.606	0.565	0.507	0.058	0.000	0.000	0.000
	Al1–Al4	2.515	1.734	0.730	1.004	0.000	0.000	0.000
	Al2–Al3	2.515	1.734	0.730	1.004	0.000	0.000	0.000
	Al2–Al4	3.604	0.565	0.507	0.058	0.000	0.000	0.000
	Al3–Al4	2.582	0.949	0.803	0.145	0.000	0.000	0.000
Ge_4^{2+}	Ge1–Ge2	2.404	1.050	0.618	0.432	0.000	0.000	0.000
	Ge1–Ge3	3.400	0.787	0.430	0.357	0.000	0.000	0.000
	Ge1–Ge4	2.404	1.050	0.618	0.432	0.000	0.000	0.000
	Ge2–Ge3	2.404	1.050	0.618	0.432	0.000	0.000	0.000
	Ge2–Ge4	3.400	0.787	0.430	0.357	0.000	0.000	0.000
	Ge3–Ge4	2.404	1.050	0.618	0.432	0.000	0.000	0.000
P_5^-	P1–P2	2.090	1.410	0.938	0.472	0.000	0.000	0.000
	P1–P3	3.382	0.114	0.046	0.068	0.000	0.000	0.000
	P1–P4	3.382	0.114	0.046	0.068	0.000	0.000	0.000
	P1–P5	2.090	1.410	0.938	0.472	0.000	0.000	0.000
	P2–P3	2.090	1.410	0.938	0.472	0.000	0.000	0.000
	P2–P4	3.381	0.114	0.046	0.068	0.000	0.000	0.000
	P2–P5	3.381	0.114	0.046	0.068	0.000	0.000	0.000
	P3–P4	2.089	1.411	0.938	0.473	0.000	0.000	0.000
	P3–P5	3.381	0.114	0.046	0.068	0.000	0.000	0.000
	P4–P5	2.090	1.410	0.938	0.472	0.000	0.000	0.000
Hf_3	Hf1–Hf2	2.676	2.073	0.739	0.889	0.444	0.000	0.000
	Hf1–Hf3	2.676	2.073	0.739	0.889	0.445	0.000	0.000
	Hf2–Hf3	2.676	2.072	0.739	0.889	0.444	0.000	0.000
Ta_3^-	Ta1–Ta2	2.451	2.168	0.818	0.965	0.169	0.000	0.216
	Ta1–Ta3	2.465	2.157	0.838	0.974	0.276	0.000	0.068
	Ta2–Ta3	2.450	2.168	0.819	0.965	0.169	0.000	0.216
$Re_2Cl_8^{2-}$	Re1–Re2	2.145	2.562	0.754	1.267	0.541	0.000	0.000
U_2	U1–U2	2.430	3.257	1.120	2.001	0.136	0.000	0.000
Th_3^{10+}	Th1–Th2	3.989	0.447	0.002	0.001	0.000	0.000	0.445
	Th1–Th3	3.989	0.447	0.002	0.001	0.000	0.000	0.445
	Th2–Th3	3.989	0.447	0.002	0.001	0.000	0.000	0.444
Th_3^{4+}	Th1–Th2	3.989	1.506	0.937	0.507	0.000	0.000	0.062
	Th1–Th3	3.989	1.506	0.937	0.507	0.000	0.000	0.062
	Th2–Th3	3.989	1.506	0.937	0.507	0.000	0.000	0.062
Th_3	Th1–Th2	3.989	1.547	0.583	0.951	0.013	0.000	0.001
	Th1–Th3	3.989	1.547	0.583	0.951	0.013	0.000	0.001
	Th2–Th3	3.989	2.124	0.556	1.391	0.177	0.000	0.001

S4 Spectral correction of the MP2 densities and its residuals

Relaxed MP2 one-particle densities are not strictly N-representable: in the full NAO basis (522 functions) each density channel of the benzene checkpoints carries a few natural occupations below zero and a few above the maximum occupation η (Table S2). Before the bond-order analysis of the main text these densities were preprocessed with the runEDDB option `--force-nrep`, which replaces the natural occupations n_k of each channel by their Euclidean projection onto

the N-representable set,

$$n_k^{\mathrm{corr}} = \min\{\max\{n_k - \mu,\, 0\},\, \eta\}, \qquad \sum_k n_k^{\mathrm{corr}} = N, \tag{S1}$$

with the natural orbitals unchanged and μ determined by bisection from the trace condition. The target electron counts are those of the input files (Gaussian checkpoint headers): $N = 42$ with $\eta = 2$ for the spin-summed RMP2 density, $N_\alpha = N_\beta = 21$ with $\eta = 1$ for the singlet UMP2 spin blocks, and $N_\alpha = 22$, $N_\beta = 20$ for the triplet UMP2 spin blocks; the program sets its target from the rounded clamped trace, which was verified to coincide with these counts in every case. The correction is a preprocessing convention for these approximate densities; it is not part of the bond-order definition, does not recover the exact MP2 density, and does not preserve all orbital or atomic populations. It also does not remove the residual effect of the subsequent quasi-degenerate grouping of the layer construction (consecutive sorted occupations differing by less than 10^{-6} form one group; groups with mean occupation below 10^{-6} are discarded), which is reported separately. Three densities are therefore distinguished below: $\mathbf{D}_{\mathrm{input}}$ (the relaxed MP2 density as read), $\mathbf{D}_{\mathrm{corr}}$ (after Eq. S1), and $\mathbf{D}_{\mathrm{rep}}$ (the density actually represented by the retained quasi-degenerate layers of $\mathbf{D}_{\mathrm{corr}}$). The corrected density written by runEDDB and the one rebuilt by the auxiliary script of Section S6 from the input blocks agree to 3×10^{-9}.

Table S2. Spectral correction of the benzene MP2 densities (full NAO basis, per density channel). N: target electron count; $n_{\min}$, $n_{\max}$: extreme input occupations; $\# < 0/\# > \eta$: numbers of input occupations outside $[0, \eta]$ (beyond 10^{-8}); μ: shift of Eq. (S1); $\Delta n_{\max}$: largest change of a single occupation; $\|\Delta\mathbf{D}\|_F$, $\Delta p_{\max}$, $\Delta q_{\max}$: Frobenius norm of the density change and largest changes of an NAO population and of an atomic population (electrons) for the step input→corr; the last three columns give the residual of the layer grouping corr→rep (Frobenius norm and discarded weight) and, for comparison, the trace drift that the same grouping produces when it is applied to the uncorrected density (where the discarded groups carry negative occupations).

	channel	N	$n_{\min}$	$n_{\max}$	$\# < 0/\# > \eta$	μ	$\Delta n_{\max}$	$\|\Delta\mathbf{D}\|_F$	$\Delta p_{\max}$	$\Delta q_{\max}$	$\|\Delta\mathbf{D}\|_F^{\mathrm{rep}}$	disc.	drift^{uncorr}
S_0 RMP2	total	42	-1.1×10^{-5}	2.0000019	6/6	8.4×10^{-8}	1.1×10^{-5}	2.2×10^{-5}	2.6×10^{-6}	2.1×10^{-6}	8.8×10^{-5}	0	4.9×10^{-5}
S_0 UMP2	alpha	21	-5.6×10^{-6}	1.0000010	6/6	4.2×10^{-8}	5.6×10^{-6}	1.1×10^{-5}	1.3×10^{-6}	1.1×10^{-6}	6.6×10^{-5}	0	2.4×10^{-5}
	beta	21	-5.6×10^{-6}	1.0000010	6/6	4.2×10^{-8}	5.6×10^{-6}	1.1×10^{-5}	1.3×10^{-6}	1.1×10^{-6}	6.6×10^{-5}	0	2.4×10^{-5}
T_1 UMP2	alpha	22	-9.5×10^{-3}	1.0000016	10/6	3.8×10^{-5}	9.5×10^{-3}	9.5×10^{-3}	1.6×10^{-3}	4.8×10^{-4}	3.6×10^{-6}	$< 10^{-12}$	1.0×10^{-2}
	beta	20	-2.0×10^{-2}	1.0000507	8/7	1.4×10^{-4}	2.0×10^{-2}	2.1×10^{-2}	5.7×10^{-3}	3.2×10^{-3}	1.5×10^{-6}	$< 10^{-12}$	2.5×10^{-2}

For the sum $\mathbf{D}_{\text{corr}} = \mathbf{P}^{\alpha}_{\text{corr}} + \mathbf{P}^{\beta}_{\text{corr}}$ of the triplet, which enters the total-density construction with $\eta = 2$, the grouping residual is $\|\Delta\mathbf{D}\|_F = 2.7 \times 10^{-5}$ with a discarded weight of 1.3×10^{-4} electrons (largest NAO-population change 1.7×10^{-6}). For the triplet the correction moves at most 0.020 in a single occupation (β block); the largest changes of an NAO population are 1.6×10^{-3} (α) and 5.7×10^{-3} (β), i.e. 7.4×10^{-3} for the spin-summed density, and those of an atomic population 4.8×10^{-4} (α) and 3.2×10^{-3} (β), i.e. 3.7×10^{-3} for the sum (C5). For the singlet the NAO- and atomic-population changes of the step input→corr are below 10^{-5} in every channel; the grouping residuals are listed separately in Table S2.

Table S3 compares the ring sums obtained from the same densities without and with the spectral correction. Both columns were obtained with the same corrected program (complete-frame trace); the "uncorrected" entries are not an exact analysis of $\mathbf{D}_{\text{input}}$ either, because the layer construction still groups near-degenerate occupations and discards layers with mean occupation below 10^{-6} (including the negative ones), so that the represented trace exceeds the electron count by 4.9×10^{-5} (singlet) and by 0.036 electrons (triplet).

Table S3. Benzene ring sums (six C–C bonds) from the relaxed MP2 densities analysed without (left) and with (right) the spectral correction of Eq. (S1); rest $= \delta + \varphi + \text{other}$. [a]Auxiliary Python evaluation (Section S6); all other rows from runEDDB.

	uncorrected				corrected			
Density, construction	ΣW	$\Sigma\sigma$	$\Sigma\pi$	rest	ΣW	$\Sigma\sigma$	$\Sigma\pi$	rest
S_0 RMP2, spin-summed layers	8.362	5.702	2.643	0.017	8.362	5.702	2.643	0.017
S_0 UMP2, per-spin layers	8.362	5.702	2.643	0.017	8.362	5.702	2.643	0.017
T_1 UMP2, per-spin layers	7.784	5.712	2.042	0.030	7.780	5.711	2.040	0.029
T_1 UMP2, total-density[a]	7.806	5.713	2.064	0.030	7.794	5.711	2.054	0.029

The ORCA RI-MP2 controls (dhf-QZVPP, relaxed densities from an RHF and from a UHF reference, read as spinless densities because the ORCA JSON files carry no α/β MP2 blocks) give, after the same correction ($\eta = 2$, $N = 42$), ring sums of 8.362/5.703/2.643/0.017 (RHF reference) and 8.362/5.702/2.643/0.017 (UHF reference) for $\Sigma W/\Sigma\sigma/\Sigma\pi/\text{rest}$, in agreement with the Gaussian values to the printed precision except for $\Sigma\sigma$ of the RHF-reference control (5.703 vs 5.702); they test the program independence of the spinless layer construction, not explicit correlated spin blocks.

S5 Full channel resolution of the benzene ring sums

Table S4 gives the complete channel resolution of the entries of Table 1 of the main text: $\Sigma W = \Sigma\sigma + \Sigma\pi + \Sigma\delta + \Sigma\varphi + \Sigma\,\text{other}$ before rounding. The φ weights are carried entirely by components below the 10^{-4} export cutoff (largest single φ-labeled weight in the final series: 3.6×10^{-5}, S_0 UMP2, a Rydberg f-type component of a ring C–C pair); "other" is the weight of coupling-frame vectors not assigned to a single channel by the 60 % purity criterion.

S6 Auxiliary Python evaluation of the total-density construction

For spin-resolved input runEDDB always evaluates the per-spin operator $2(\mathbf{\Omega}^{\alpha} + \mathbf{\Omega}^{\beta})$; the "total-density" entries of Table 1 of the main text (the $\eta = 2$ layer construction applied to $\mathbf{D} = \mathbf{P}^{\alpha} + \mathbf{P}^{\beta}$) are not available as a program option and were evaluated with an auxiliary Python script (`nba_independent.py` and `final_mp2_analysis.py`, provided with the numerical material). The script reads the original checkpoint densities and the complete NAO set $\mathbf{T}$ exported by runEDDB for the same run, forms $\mathbf{D}_{\text{NAO}} = \mathbf{T}^{-1}\mathbf{D}_{\text{AO}}\mathbf{T}^{-\top}$ (the NAO set is square and orthonormal,

Table S4. Complete channel resolution of the benzene ring sums (six C–C bonds, def2-QZVPP, full NAO basis). MP2 rows: spectrally corrected densities. [a]Auxiliary Python evaluation on the same (corrected) blocks; all other rows from the runEDDB diagnostic files.

Wavefunction	Treatment	ΣW	$\Sigma\sigma$	$\Sigma\pi$	$\Sigma\delta$	$\Sigma\varphi$	Σ other
Ground-state singlet S_0							
RHF	closed shell	8.642	5.865	2.777	0.000	0.000	0.000
UHF	per-spin	8.642	5.865	2.777	0.000	0.000	0.000
RMP2 (corr.)	spin-summed layers	8.362	5.702	2.643	0.005	0.000	0.011
UMP2 (corr.)	per-spin layers	8.362	5.702	2.643	0.005	0.000	0.011
Lowest triplet T_1							
UHF	per-spin	7.660	5.875	1.784	0.000	0.000	0.001
	total-density[a]	7.797	5.879	1.918	0.000	0.000	0.001
ROHF	per-spin	7.944	5.881	2.063	0.000	0.000	0.000
	total-density[a]	7.944	5.881	2.063	0.000	0.000	0.000
UMP2 (corr.)	per-spin layers	7.780	5.711	2.040	0.006	0.000	0.024
	total-density[a]	7.794	5.711	2.054	0.005	0.000	0.024

$\mathbf{T}^\top\mathbf{S}\mathbf{T} = \mathbf{I}$, so that $\mathbf{S} = \mathbf{T}^{-\top}\mathbf{T}^{-1}$), applies the spectral correction of Eq. (S1) to each spin block with the electron counts of the input, and then re-implements, in the same NAO basis, the closed-shell, per-spin, and cumulative-layer bond-order operators (same grouping thresholds), the complete coupling frame of $\mathbf{M}(\mathbf{D}_{AB})$, the projected weights, the branch assignment, and the $(\hat{\mathbf{n}}\cdot\mathbf{L})^2$ angular classifier with the same purity threshold. Its validation consists of reproducing the native results of the program: for all seven benzene densities the per-pair bond indices (operator traces) agree with the runEDDB diagnostic files to 1.6×10^{-8} (the precision of the exchanged formatted files), the corrected spin blocks agree with the corrected density written by runEDDB to 3×10^{-9}, and none of the tested per-pair channel sums (σ, π, δ, φ, other; 642 cells for the uncorrected and 462 cells for the corrected series) differs from the program value after rounding to three decimals. Differences of up to 8×10^{-5} appear at the fifth decimal in the C–H channel sums of the MP2 densities, where individual labels in nearly degenerate Rydberg subspaces depend on the eigensolver; this is the expected basis dependence of labels inside degenerate subspaces (Section 5 of the main text), not a difference of the operators. The total-density and per-spin entries of the triplet therefore start from the same corrected blocks and use the same representation; they differ only in the operator construction.

S7 Analytic comparison of the three orbital representations

This section works out, for one small open-shell determinant, the three representations discussed in Section 3 of the main text: separate spin-resolved bond-order orbitals, the eigenvectors of the combined operator $\mathbf{\Omega}$, and the projection onto the complete coupling frame of the total density. It is an analytic model with three orthonormal atomic functions and is not a result for any real molecule; it illustrates the algebra only.

Let $\{\chi_{A1}, \chi_{A2}, \chi_B\}$ be an orthonormal basis with two functions on atom A and one on atom B, and let one electron of each spin occupy

$$u_\alpha = \frac{\chi_{A1}+\chi_B}{\sqrt{2}}, \qquad u_\beta = \frac{\chi_{A2}+\chi_B}{\sqrt{2}}, \qquad \mathbf{P}^\alpha = u_\alpha u_\alpha^\top, \quad \mathbf{P}^\beta = u_\beta u_\beta^\top.$$

Both spin projectors are idempotent, so this is a single determinant with one α and one β electron (it is not a spin-pure singlet). The A–B blocks are $\mathbf{P}^\alpha_{AB} = (1/2,\,0)^\top$ and $\mathbf{P}^\beta_{AB} = (0,\,1/2)^\top$, each with a single singular value $1/2$, so that the open-shell operator of the main text, $\mathbf{\Omega} = 2(\mathbf{\Omega}^\alpha+\mathbf{\Omega}^\beta)$,

is

$$\boldsymbol{\Omega} = \frac{1}{4}\begin{pmatrix} 1 & 0 & 1 \\ 0 & 1 & 1 \\ 1 & 1 & 2 \end{pmatrix}, \qquad \operatorname{Tr}\boldsymbol{\Omega} = 1 = 2\big(\|\mathbf{P}^{\alpha}_{AB}\|_F^2 + \|\mathbf{P}^{\beta}_{AB}\|_F^2\big),$$

in the ordering $(\chi_{A1}, \chi_{A2}, \chi_B)$. The spin-summed density $\mathbf{D} = \mathbf{P}^{\alpha} + \mathbf{P}^{\beta}$ has the block $\mathbf{D}_{AB} = (1/2,\ 1/2)^{\top}$, and the direct quadratic contraction $\|\mathbf{D}_{AB}\|_F^2 = 1/2$ is not the open-shell index. Table S5 lists the three representations.

Table S5. Three representations of the bond-order operator of the analytic model. Coefficients refer to $(\chi_{A1}, \chi_{A2}, \chi_B)$; weights are $2\langle u_\varsigma|\boldsymbol{\Omega}^\varsigma|u_\varsigma\rangle$ for the spin orbitals, eigenvalues for the eigenvectors of $\boldsymbol{\Omega}$, and projected weights $n_k = \langle \mathbf{q}_k|\boldsymbol{\Omega}|\mathbf{q}_k\rangle$ for the coupling frame (λ_k: eigenvalue of $\mathbf{M}(\mathbf{D}_{AB})$).

Representation	Vector	λ_k	Weight
spin-resolved BOOs	$u_\alpha = (\chi_{A1} + \chi_B)/\sqrt{2}$	–	$1/2$
	$u_\beta = (\chi_{A2} + \chi_B)/\sqrt{2}$	–	$1/2$
eigenvectors of $\boldsymbol{\Omega}$	$\mathbf{z}_1 = (\chi_{A1} + \chi_{A2} + 2\chi_B)/\sqrt{6}$	–	$3/4$
	$\mathbf{z}_2 = (\chi_{A1} - \chi_{A2})/\sqrt{2}$	–	$1/4$
coupling frame of $\mathbf{M}(\mathbf{D}_{AB})$	$\mathbf{q}_+ = (\chi_{A1} + \chi_{A2})/2 + \chi_B/\sqrt{2}$	$+1/\sqrt{2}$	$(3 + 2\sqrt{2})/8 \approx 0.729$
	$\mathbf{q}_0 = (\chi_{A1} - \chi_{A2})/\sqrt{2}$	0	$1/4$
	$\mathbf{q}_- = (\chi_{A1} + \chi_{A2})/2 - \chi_B/\sqrt{2}$	$-1/\sqrt{2}$	$(3 - 2\sqrt{2})/8 \approx 0.021$

All three sets of weights sum to $\operatorname{Tr}\boldsymbol{\Omega} = 1$. The spin-resolved orbitals u_α and u_β are not mutually orthogonal (overlap $1/2$) and keep the spin information separate. The eigenvectors $\mathbf{z}_1, \mathbf{z}_2$ give the exact spectral decomposition of $\boldsymbol{\Omega}$. The coupling frame $\{\mathbf{q}_+, \mathbf{q}_0, \mathbf{q}_-\}$ is the complete eigenbasis of the spinless coupling matrix of the total density; its projected weights preserve the trace, but $\boldsymbol{\Omega}$ is not diagonal in this frame: $\langle \mathbf{q}_+|\boldsymbol{\Omega}|\mathbf{q}_-\rangle = -1/8$, so the diagonal weights do not reconstruct the full operator, and the negative branch $\mathbf{q}_-$ carries a positive weight although its coupling eigenvalue is negative. In the closed-shell limit ($\mathbf{P}^{\alpha} = \mathbf{P}^{\beta}$) the off-diagonal element and the negative-branch weight vanish and the three representations coincide. The example shows the three representations as complementary reporting choices of one operator; it does not establish a chemical advantage of any of them.

S8 Input files and geometries used

For each system the descriptive line gives the charge, spin multiplicity and the level of theory; the geometry that follows is the one used for the reported density.

S8.1 All-metal and inorganic clusters (PBEh-3c)

Li_4Al_4 (rectangular Al_4^{4-} core, neutral, singlet)

Li4Al4.inp

```
! PBEh-3c TightSCF
* xyz 0 1
  Al     1.29153786    1.25758331    0.00008275
  Al    -1.29132484    1.25691468    0.00016654
  Al    -1.29135738   -1.25855736    0.00000024
  Al     1.29079324   -1.25784251    0.00023105
  Li     0.00017697    0.00157536    2.20102801
  Li     0.00034709    0.00160849   -2.20051683
  Li     3.55212694   -0.00089368    0.00081700
  Li    -3.55229988   -0.00036828    0.00083123
*
```

Li_2Al_4 (square Al_4^{2-} core, neutral, singlet)

Li2Al4.inp

```
! PBEh-3c TightSCF
* xyz 0 1
  Al     1.29354874    1.29444476    0.00000042
  Al    -1.29405652    1.29445405    0.00000033
  Al    -1.29400850   -1.29310848   -0.00000105
  Al     1.29347044   -1.29306890   -0.00000049
  Li     0.00051152   -0.00133779    2.31472806
  Li     0.00052044   -0.00135196   -2.31472455
*
```

Ge_4^{2+} (square, dication, singlet)

Ge4_2+.inp

```
! PBEh-3c TightSCF
* xyz 2 1
  Ge     1.20198     1.20198     0.00001
  Ge    -1.20198     1.20198    -0.00001
  Ge    -1.20198    -1.20198     0.00001
  Ge     1.20198    -1.20198    -0.00001
*
```

cyclo-P_5^- (planar pentagon, anion, singlet)

P5-.inp

```
! PBEh-3c TightSCF
* xyz -1 1
  P     -0.00000     1.77798     0.00006
  P     -1.69051     0.54916    -0.00001
  P     -1.04465    -1.43816    -0.00005
  P      1.04465    -1.43816     0.00008
  P      1.69051     0.54916    -0.00009
*
```

$Re_2Cl_8^{2-}$ (eclipsed D_{4h}, dianion, singlet; Re–Re $= 2.145$ Å)

Re2Cl8_2-.inp

```
! PBEh-3c TightSCF
* xyz -2 1
  Re    -0.00000    -0.00000     1.07232
  Re    -0.00000     0.00000    -1.07231
  Cl     2.27305    -0.00002     1.66642
  Cl     0.00002     2.27305     1.66642
  Cl    -2.27305     0.00002     1.66642
  Cl    -0.00002    -2.27305     1.66642
  Cl     2.27306     0.00002    -1.66642
  Cl    -0.00002     2.27306    -1.66642
  Cl    -2.27306    -0.00002    -1.66642
  Cl     0.00003    -2.27306    -1.66642
*
```

Hf_3 ($^1A_1'$ equilateral triangle, neutral, singlet; Hf–Hf $= 2.676$ Å)

Hf3.inp

```
! PBEh-3c TightSCF
* xyz 0 1
  Hf     1.54486     0.00019     0.00000
  Hf    -0.77260     1.33804     0.00000
  Hf    -0.77226    -1.33823     0.00000
*
```

Ta_3^- (quintet, near-equilateral; Ta–Ta ≈ 2.46 Å)

Ta3-.inp

```
! PBEh-3c TightSCF
* xyz -1 5
  Ta     1.42037    0.00475    0.00000
  Ta    -0.70607    1.22283    0.00000
  Ta    -0.71430   -1.22758    0.00000
*
```

S8.2 Actinide systems (PBE0/SARC-DKH-TZVP, DKH2)

The Th_3 ring is held at the fixed experimental geometry across the charge series; only the electron count changes.

Th_3^{10+} (2 ring electrons, singlet)

Th3_10+.inp

```
! PBE0 D4 SARC-DKH-TZVP DKH2 RIJCOSX SARC/J defgrid2
* xyz 10 1
  Th     2.30329    0.00000    0.00000
  Th    -1.15164    1.99474    0.00000
  Th    -1.15164   -1.99474    0.00000
*
```

Th_3^{4+} (8 ring electrons, singlet)

Th3_4+.inp

```
! PBE0 D4 SARC-DKH-TZVP DKH2 RIJCOSX SARC/J defgrid2
* xyz 4 1
  Th     2.30329    0.00000    0.00000
  Th    -1.15164    1.99474    0.00000
  Th    -1.15164   -1.99474    0.00000
*
```

Th_3 (neutral, singlet)

Th3_0.inp

```
! PBE0 D4 SARC-DKH-TZVP DKH2 RIJCOSX SARC/J defgrid2
* xyz 0 1
  Th     2.30329    0.00000    0.00000
  Th    -1.15164    1.99474    0.00000
  Th    -1.15164   -1.99474    0.00000
*
```

U_2 (septet, literature bond length 2.43 Å)

U2.inp

```
! PBE0 D4 SARC-DKH-TZVP DKH2 RIJCOSX SARC/J defgrid2
%scf maxiter 500 end
* xyz 0 7
  U     0.00000    0.00000    0.00000
  U     0.00000    0.00000    2.43000
*
```

S9 HPC resources

All calculations were performed using the supercomputer Helios available in the HPC infrastructure PLGrid (ACK Cyfronet AGH, grant no. PLG/2025/018981) and an in-house server with the

following specification:

- Model: Supermicro Hyper A+ Server AS-2125HS-TNR.
- Operating system: Debian GNU/Linux x86_64.
- RAM: 24×64 GB DDR5-4800 MHz ECC REG.
- CPU: 2× AMD EPYC 9554 (Genoa), 64C/128T, 3.1 GHz, 256 MB L3.
- NVMe (system): Intel Optane DC P5800X 3.2 TB PCIe 4.0.
- NVMe (scratch): Micron 9400 MAX 12.8 TB PCIe 4.0.
- Network: Supermicro AIOM 2×10 GbE.
